# Health risk assessment and removal of nitrate and sulfate ions using Mg/Al quartz-based geopolymer: a case study from Siwa Oasis

**DOI:** 10.1186/s13065-026-01721-4

**Published:** 2026-01-16

**Authors:** Mohamed Hamdy Eid, May Bin-Jumah, Amira S. Diab, Attila Kovács, Péter Szűcs, Bashir Salah, Ahmed Mehaney, Mostafa R. Abukhadra

**Affiliations:** 1 Faculty of Earth Science, Institute of Environmental Management, University of Miskolc, Miskolc- Egyetemváros, 3515 Hungary; 2 Geology Department, Faculty of Science, Beni-Suef University, Beni-Suef, 65211 Saudi Arabia; 3 Department of Biology, College of Science, Princess Nourah Bint Abdulrahman University, P.O. BOX 84428, Riyadh, 11671 Egypt; 4 Department of Chemistry, Faculty of Science, Beni-Suef University, Beni-Suef, Egypt; 5 Department of Industrial Engineering, College of Engineering, King Saud University, Riyadh, 12372 Saudi Arabia; 6 Physics Department, Faculty of Science, Beni-Suef University, Beni-Suef, 62512 Egypt; 7 Geosciences Department, College of Science, United Arab Emirates University, Al Ain, 15551 United Arab Emirates

**Keywords:** Siwa oasis, Groundwater contamination, Sulfate removal, Nitrate removal, Geopolymer adsorption, Water treatment

## Abstract

This study assesses the health risks associated with nitrate and sulfate contamination in surface and groundwater sources within the Siwa Oasis, located in Egypt’s northwestern desert. It also explores the effectiveness of a low-cost magnesium/aluminum quartz-based geopolymer (Mg/GP) as an enhanced adsorbent for contaminant removal. Nitrate concentrations reached up to 29.4 mg/L, exceeding acceptable safety limits, as indicated by a total hazard quotient (THQ) greater than 1. This level of exposure presents significant health risks, particularly for children (oral HQ up to 1.23). Sulfate concentrations also surpassed irrigation standards, further highlighting concerns regarding water quality in the region. To mitigate these risks, Mg/GP was synthesized as a modified, cost-effective geopolymer exhibiting high adsorption capacities for both nitrate (166.1 mg/g) and sulfate (234.1 mg/g) ions. The adsorption behavior followed pseudo-first-order kinetics (R² > 0.91) and was well-described by the Langmuir isotherm model (R² > 0.99). Additionally, statistical physics-based equilibrium modeling was employed to analyze steric and energetic adsorption characteristics. The analysis of active site density revealed a higher saturation capacity for sulfate (Nm = 145.4 mg/g) compared to nitrate (Nm = 18.8 mg/g), indicating more efficient sulfate uptake. However, nitrate ions demonstrated a stronger aggregation affinity (*n* = 8.83) than sulfate (*n* = 1.6), with both values exceeding 1, suggesting the presence of multi-ionic interaction mechanisms. These interactions were predominantly physical and exothermic, as evidenced by adsorption energies (ΔE < 20 kJ/mol). Field application of Mg/GP for groundwater treatment confirmed its practical potential, achieving a 72.4% reduction in sulfate concentration (initially 456.2 mg/L) and a 94.2% reduction in nitrate concentration (initially 10.4 mg/L).

**Supplementary Information:**

The online version contains supplementary material available at 10.1186/s13065-026-01721-4.

## Introduction

The contamination of freshwater resources by chemical pollutants poses a serious and growing threat to both environmental sustainability and public health, sparking widespread concerns about the long-term security and quality of global water supplies [1]. One of the primary causes of this issue is the unchecked discharge of highly polluted wastewater from industrial, agricultural, and mining activities, which has had far-reaching ecological consequences. Among the most frequently reported pollutants in surface and groundwater systems are sulfate and nitrate ions, which often appear in elevated concentrations that demand immediate attention [2].

High sulfate levels can disrupt the natural sulfur cycle and are associated with a range of health problems, especially with long-term exposure [3–5]. These include gastrointestinal issues such as diarrhea and dehydration, as well as more severe conditions like cardiovascular disease, sensory impairments, and reproductive disorders. In some cases, sulfate exposure has been linked to changes in methemoglobin and sulfhemoglobin levels, which can have serious physiological effects [5]. Recognizing these risks, many countries have set strict regulatory limits for sulfate concentrations in drinking water—typically between 250 and 500 mg/L, depending on the intended use. The World Health Organization (WHO) also recommends that sulfate levels not exceed 250 mg/L in drinking water to ensure consumer safety [5–8].

Nitrate contamination has similarly emerged as a critical environmental and health concern, particularly in its soluble ionic form. Concentrations above 10 mg/L are associated with the formation of carcinogenic nitrosamines and the risk of methemoglobinemia, commonly known as “blue baby syndrome”—a potentially fatal condition affecting infants [9–12]. Because of these risks, nitrate levels in water are closely monitored, and a wide range of treatment technologies have been developed to meet drinking water standards.

Technologies such as adsorption, flocculation, membrane separation, ion exchange, and nanofiltration have shown strong potential for removing sulfate and nitrate from water sources [13–15]. Among these, adsorption has gained particular attention due to its simplicity, cost-effectiveness, and high removal efficiency, as well as its ability to allow material recovery and reuse [16–18]. Recent research has demonstrated that many nanomaterials used in adsorption are both affordable and environmentally sustainable, making them attractive options for real-world water treatment [19, 20]. The performance of any adsorbent, however, depends on several key factors, including its production cost, the synthesis process, material availability, adsorption capacity, selectivity, reusability, and overall chemical and physical properties [21, 22]. For this reason, researchers have increasingly focused on developing adsorbents from low-cost, naturally available materials [23, 24].

In particular, terrestrial materials such as natural rocks and minerals have drawn increasing interest because of their abundance and favorable environmental profile [25]. One such class of materials is aluminosilicate-based geopolymers, which have demonstrated great promise in water purification applications [26]. These geopolymers are formed through the polymerization of AlO₄ and SiO₄ units into a stable three-dimensional network. This structure imparts excellent porosity, high surface area, strong surface reactivity, rapid adsorption kinetics, and good ion-exchange capacity, making them highly suitable for removing contaminants from water [26–28].

Despite these advantages, conventional geopolymers face limitations that hinder their practical application. For instance, they often lack sufficient external active sites, which limits their ability to adsorb dissolved ions effectively [29]. They also tend to have lower mechanical strength and smaller surface areas than other porous materials, which restricts their use in large-scale systems [30–32]. To overcome these drawbacks, researchers have been exploring ways to modify geopolymers and improve their performance. This includes using alternative precursors such as fly ash, kaolinite, zeolite, and even industrial and agricultural wastes to enhance textural and structural properties [30–32]. Ultimately, the effectiveness of a geopolymer depends heavily on the specific raw materials used in its synthesis [33].

To boost their efficiency for sulfate and nitrate removal, recent studies have focused on incorporating advanced precursors like nano-quartz as a silica source and magnesium–aluminum layered double hydroxides (Mg/Al LDHs) as an alumina source. These components not only improve the surface area and ion-exchange capacity of geopolymers but also introduce beneficial functional groups such as Mg²⁺, K⁺, and Na⁺. One of the key challenges in conventional geopolymer synthesis is the use of synthetic silica, which is expensive and not environmentally friendly [34, 35]. Moreover, synthetic silica often lacks the desirable surface and electrical properties that are important for adsorption. In contrast, natural silica—especially quartz (SiO₂), the most abundant mineral in the Earth’s crust—offers excellent chemical and mechanical stability, is low in cost, and is widely available, making it a highly attractive substitute [36, 37]. Replacing synthetic silica with natural quartz improves both the environmental sustainability and cost-effectiveness of the overall synthesis process [38].

Based on these insights, this study aims to develop a novel geopolymer enriched with magnesium and aluminum, using Mg/Al LDH and natural quartz as the primary raw materials. The synthesized geopolymer was tested as an adsorbent for the removal of sulfate (SO₄²⁻) and nitrate (NO₃⁻) ions from contaminated water. Key experimental parameters were examined, and adsorption performance was assessed using advanced equilibrium modeling based on statistical physics. This included a detailed analysis of steric parameters—such as saturation capacity and site occupancy—and energetic factors like adsorption energy, internal energy, enthalpy, and entropy. To confirm its real-world effectiveness, the material was applied to the treatment of actual groundwater samples collected from the Siwa Oasis in Egypt. In light of the growing global demand for low-cost, scalable, and efficient water treatment solutions, this study offers valuable contributions to the field of water remediation. The findings highlight the strong potential of geopolymer-based adsorbents and provide a practical pathway for mitigating sulfate and nitrate pollution in real-world environmental settings.

## Site description

**Fig. 1:**
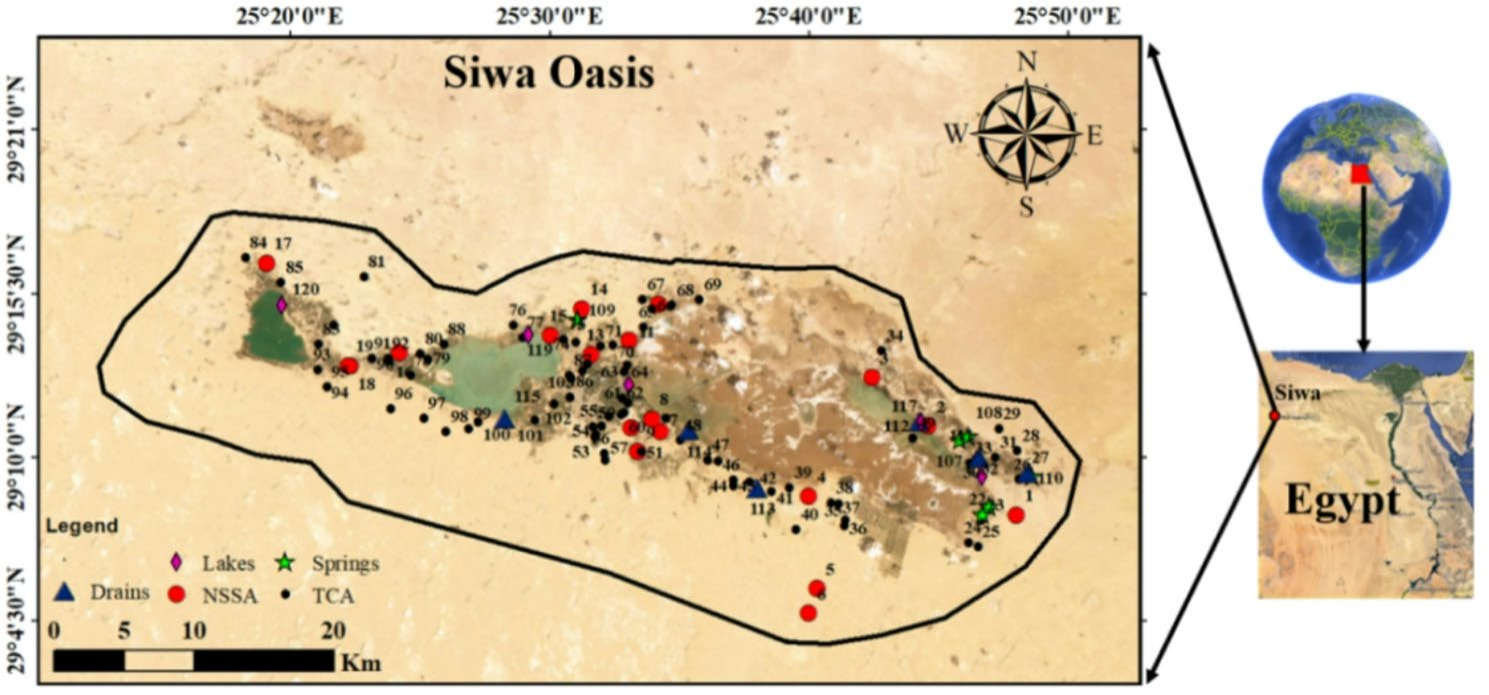
Location of sampling points including NSSA, TCA, springs, Drains and salt lakes

Siwa Oasis, situated in Egypt’s northern Western Desert, spans an area of approximately 1,100 square kilometers. Located around 320 km south of the Mediterranean coast, the oasis is home to an estimated population of 23,000 residents [39] (Fig. 1). The local economy is primarily driven by agriculture, with date palm cultivation, olive oil production, and the farming of various fruits and vegetables forming the economic backbone. Additionally, small-scale industrial activities—such as mineral water bottling and olive oil processing—play a vital role in supporting the region’s economic growth [39, 40].

The oasis experiences a harsh arid climate characterized by extremely high evaporation rates, reaching up to 16.8 mm/day during the summer and dropping to about 5.4 mm/day in the winter. Annual precipitation is exceptionally low, averaging only 10 mm [41, 42]. These challenging climatic conditions, combined with geographic isolation and limited freshwater resources, pose significant constraints on both livelihoods and sustainable development in the region.

## Hydrogeology of study region

The geological framework of Siwa Oasis encompasses a range of hydrostratigraphic units. Near the surface, Quaternary deposits—including sand dunes and salt flats—dominate, extending to shallow depths. Beneath these layers lie the Miocene and Eocene formations, which collectively form the Tertiary Carbonate Aquifer (TCA). This aquifer system is primarily composed of limestone and dolomite, interbedded with thin layers of shale and clay. Deeper strata include Mesozoic formations such as the Lower Cretaceous Nubian Sandstone and Upper Cretaceous shale, as well as Paleozoic basement rocks [43]. Groundwater resources in the oasis are mainly derived from two principal aquifers: the shallow TCA and the deeper Nubian Sandstone Aquifer System (NSSA). The Miocene section of the TCA provides water for both agricultural and domestic use, while the NSSA, known for its high-quality reserves, is primarily used for drinking water and large-scale irrigation [44].

**Fig. 2:**
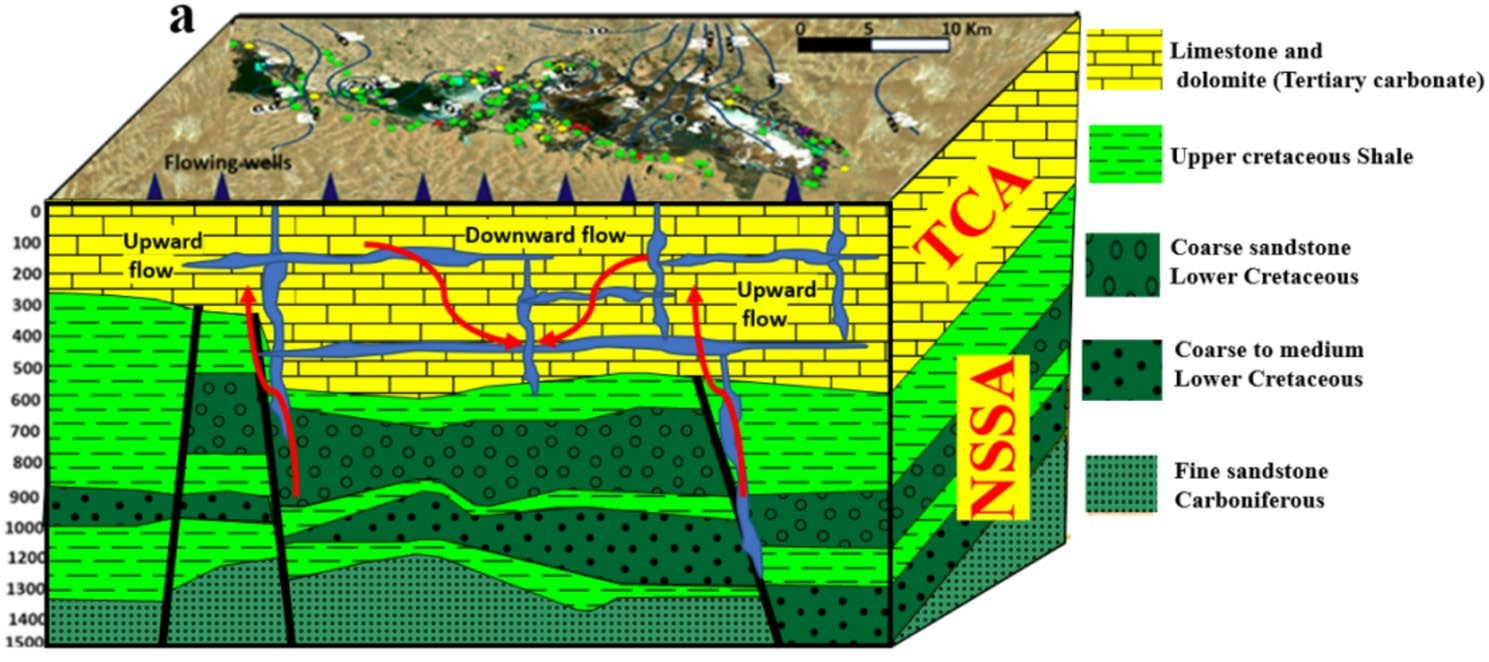
Hydrostratigraphic units of the aquifer systems and groundwater flow from deep NSSA to shallow TCA along fracture system [42]

The region faces significant environmental challenges, particularly in areas surrounding major salt lakes such as Zeitoun (eastern Siwa), Aghormi and Siwa (central Siwa), and Maraqi (western Siwa). These zones are heavily impacted by soil salinization and waterlogging, emphasizing the need for sustainable groundwater management practices [42, 44, 45]. Groundwater flow within the NSSA, based on hydraulic head measurements from 27 observation wells (Fig. 2b), generally follows southeast-to-northwest (SE–NW) and southwest-to-northeast (SW–NE) trajectories. However, intensive agricultural pumping has led to pronounced over-extraction, resulting in a notable cone of depression in the central region of the oasis. This situation highlights the urgent necessity for strategic groundwater management to balance agricultural water demands with the long-term sustainability of regional water resources.

## Experimental work

### Materials

Quartz samples were obtained from the Marwit El Sweiqat quartz mine, located in Egypt’s Eastern Desert. Following beneficiation, the quartz precursor achieved a chemical purity of 99.97%, with only trace impurities remaining. The particle size distribution ranged between 100 μm and 20 μm. The chemicals used in the synthesis of the adsorbent included magnesium nitrate hexahydrate (Mg(NO₃)₂·6 H₂O, 98%), sodium hydroxide (NaOH, 98%), aluminum nitrate nonahydrate (Al₂(NO₃)₃·9 H₂O, 98%), and potassium hydroxide (KOH, 98%). All reagents were of laboratory grade and procured from Sigma-Aldrich. Standard stock solutions of sulfate and nitrate (1000 mg/L; Sigma-Aldrich, Egypt) were used as target pollutants during the adsorption experiments.

### Synthesis of Mg/AL LDH

The Mg/Al–NO₃ layered double hydroxide (LDH) was synthesized using the co-precipitation method. A cationic solution with a molar ratio of Mg: Al = 2:1 was prepared by dissolving 23.1 g of Mg(NO₃)₂·6 H₂O and 11.25 g of Al₂(NO₃)₃·9 H₂O in 120 mL of distilled water, followed by high-speed stirring for 30 min. In parallel, an alkaline solution was prepared by dissolving 11.18 g of K₂CO₃ in 120 mL of distilled water, maintaining a KOH: K₂CO₃ molar ratio of 2:1. The alkaline solution was then added dropwise to the cationic solution under vigorous stirring (1500 rpm), maintaining the pH at 10. The resulting slurry was aged at 60 °C for 18 h under continuous stirring to ensure complete reaction and structural development.

### Synthesis of Mg/Al quartz based geopolymer (Mg/GP)

The Mg/Al-based geopolymer was synthesized in three steps. First, 20 g of the precipitated Mg/Al–LDH was dispersed in the remaining alkaline solution and stirred vigorously. Quartz powder was then added to the suspension, and the mixture was continuously stirred for 4 h to ensure homogeneity. In the second step, the mixture was transferred to a glass reactor containing 2 M KOH and stirred at 80 °C for 24 h to facilitate polymerization. The resulting solid product was separated, air-dried at room temperature for 48 h, and then thermally treated at 500 °C for 4 h. The final product was washed thoroughly to remove residual potassium and labeled as Mg/GP for use in subsequent adsorption studies.

### Characterization instruments

X-ray diffraction (XRD) analysis was carried out using a PANalytical Empyrean diffractometer to identify crystalline phases, scanning over a 2θ range of 0° to 70°. Fourier-transform infrared spectroscopy (FTIR) was performed on a Shimadzu FTIR-8400 S instrument across a wavenumber range of 400–4000 cm⁻¹ to examine functional groups. Surface morphology was observed using a Gemini Zeiss Ultra 55 scanning electron microscope (SEM) after gold sputter-coating. Specific surface area and porosity were analyzed by nitrogen adsorption–desorption isotherms using a Beckman Coulter SA3100 analyzer, following degassing of the samples.

###  Sampling and analysis

A total of 110 sampling sites were selected across the Siwa Oasis to comprehensively represent various water resources. In February 2022, 120 water samples were collected from shallow aquifers (TCA), deep aquifers (NSSA), natural springs, surface drains (6 samples each), and five salt lakes. In-situ measurements—including pH, temperature, and electrical conductivity (EC)—were recorded using calibrated portable meters. All samples were preserved at 4 °C and transported to the laboratory for analysis.

Alkali metal ions (Na⁺, K⁺) were quantified using flame photometry, while total hardness (TH) was determined by EDTA titration. Carbonate (CO₃²⁻) and bicarbonate (HCO₃⁻) levels were assessed volumetrically. Nitrate (NO₃⁻) and sulfate (SO₄²⁻) concentrations were measured via ion chromatography. Magnesium (Mg²⁺) concentrations were calculated based on total hardness and calcium (Ca²⁺) measurements. Chloride (Cl⁻) was determined by titration with silver nitrate (AgNO₃).

### 4.6. Health risks regarding nitrate

The U.S. Environmental Protection Agency (EPA) has established a comprehensive framework for assessing human health risks, particularly those related to nitrate exposure [46, 47]. In this study, health risks were evaluated for both adults and children via two exposure pathways: oral ingestion and dermal contact, using well-established equations [48]. For oral ingestion, the chronic daily intake (CDI) was calculated using Eq. (1):

1 $$\:\mathrm{C}\mathrm{D}\mathrm{I}=\frac{C\times\:\mathrm{I}\mathrm{R}\times\:\mathrm{E}\mathrm{F}}{\mathrm{A}\mathrm{B}\mathrm{W}\times\:\mathrm{A}\mathrm{E}\mathrm{T}}\:\times\:\mathrm{E}\mathrm{D}$$

Where C is the nitrate concentration (mg/L), IR is the ingestion rate (2.5 L/day for adults, 1 L/day for children), EF is the exposure frequency (365 days/year), and ED is the exposure duration (64 years for males, 67 years for females, and 12 years for children). The average body weight (ABW) is 65 kg for men, 55 kg for women, and 15 kg for children. The average exposure time (AET) is 23,360 days for males, 24,455 days for females, and 4380 days for children. The hazard quotient (HQ_oral_) is then determined using Eq. (2):

2 $$\:{HQ}_{oral}=\frac{{\mathrm{C}\mathrm{D}\mathrm{I}}_{\mathrm{o}\mathrm{r}\mathrm{a}\mathrm{l}}}{\mathrm{R}\mathrm{F}\mathrm{D}}$$

where RFD is the reference dose for nitrate (1.6 mg/kg/day) [46, 47].

For dermal exposure, the dermal absorbed dose (DAD) is calculated using Eq. (3):

3 $$\:\mathrm{D}\mathrm{A}\mathrm{D}=\frac{TC\times\:\mathrm{C}\times\:\mathrm{K}\mathrm{i}\times\:\mathrm{E}\mathrm{V}\times\:\mathrm{E}\mathrm{D}\times\:\mathrm{E}\mathrm{F}\times\:\mathrm{S}\mathrm{S}\mathrm{A}\times\:\mathrm{C}\mathrm{F}}{\mathrm{A}\mathrm{B}\mathrm{W}\times\:\mathrm{A}\mathrm{E}\mathrm{T}}\:\times\:\mathrm{E}\mathrm{D}$$

where TC is contact time (0.4 h/day), Ki is the skin adsorption coefficient (0.001 cm/hour), EV is the bathing frequency (1/day), SSA is the skin surface area (16,600 cm² for adults and 12,000 cm² for children), and CF is the conversion factor (0.001 for both adults and children). The hazard quotient for dermal exposure (HQ_dermal_ ​) is determined using Eq. (4):

4 $$\:{HQ}_{dermal}=\frac{\mathrm{D}\mathrm{A}\mathrm{D}}{\mathrm{R}\mathrm{F}\mathrm{D}}$$

The total hazard quotient (THQ), combining both oral and dermal risks, is calculated using Eq. (5):

5 $$\:THQ={\sum\:}_{i=1}^{n}(HQoral+HQdermal)$$

The THQ is a dimensionless value used to estimate the potential risk of non-carcinogenic health effects due to nitrate exposure. A THQ value greater than 1 indicates a potential health risk to individuals [49]. This risk assessment framework allows for a comprehensive understanding of nitrate’s health implications and supports the evaluation of environmental safety in contaminated regions.

### Batch adsorption experiments

Batch adsorption experiments were conducted to evaluate the removal efficiency of sulfate (SO₄²⁻) and nitrate (NO₃⁻) ions using the synthesized Mg/GP adsorbent. The experiments were designed to examine the effects of pH (ranging from 3 to 8), initial ion concentrations (25–200 mg/L), and contact time (20–400 min). All experiments were carried out in triplicate to ensure reproducibility, using a fixed solution volume of 100 mL and an adsorbent dosage of 0.3 g/L. Equilibrium adsorption studies were performed at three different temperatures: 293 K, 303 K, and 313 K. After reaching equilibrium, the mixtures were filtered to separate the Mg/GP particles, and the residual concentrations of sulfate and nitrate in the filtrates were measured. Sulfate concentrations were determined using a Dionex DX-120 ion chromatography system. Nitrate concentrations were analyzed using the same method following standard procedures. The adsorption capacity of Mg/GP was calculated using Eq. (6), based on the experimental data obtained. Calibration standards for sulfate and nitrate were obtained from Merck (Germany) and verified against standards provided by the National Institute of Standards and Technology (NIST). In the equation, Q_e_ ​ represents the equilibrium adsorption capacity (mg/g), C_0_​ and C_e​_ are the initial and equilibrium concentrations of the anions (mg/L), V is the solution volume (mL), and mmm is the mass of Mg/GP used (mg).

6 $$\:{Q}_{e\:(mg/g)}=\frac{{(C}_{o}-{C}_{e})V}{m}$$

### Conventional and modern equilibrium investigations

The adsorption behavior of sulfate (SO₄²⁻) and nitrate (NO₃⁻) ions onto the Mg/GP composite was evaluated using a combination of conventional kinetic models, classical equilibrium isotherms, and advanced adsorption models grounded in the theoretical principles of statistical physics (Table S1). Kinetic and classical equilibrium models were analyzed through non-linear regression of the experimental data, with model performance assessed using standard statistical indicators such as the coefficient of determination (*R²*, Eq. 7) and the chi-squared test (χ², Eq. 8). These parameters provided insight into the accuracy and goodness-of-fit of each model.

For the advanced isotherm investigations, adsorption data were fitted to modern statistical physics-based models to better understand the molecular-level interactions between the ions and the adsorbent surface. The quality of these fits was evaluated using the determination coefficient (*R²*) and the root mean square error (RMSE, Eq. 9), providing a more robust analysis of model reliability. In the applied models, the parameters *m′*, *p*, *Qₑ*,_*cal*_, and *Qₑ*,_*exp*_ denote the uptake data, adsorption-affecting variables (e.g., site density, interaction energy), calculated equilibrium adsorption capacities, and experimentally measured values, respectively.

7 $$\:{\mathrm{R}}^{2}=1-\frac{\sum\:({Q}_{e,\:exp}-{Q}_{e,\:cal}{)}^{2}}{\sum\:({Q}_{e,\:exp}-{Q}_{e,\:mean}{)}^{2}}$$

8 $$\:{{\upchi\:}}^{2}=\sum\:\frac{({Q}_{e,\:exp}-{Q}_{e,\:cal}{)}^{2}}{{\mathrm{Q}}_{\mathrm{e},\:\mathrm{c}\mathrm{a}\mathrm{l}}}$$

9 $$\:\mathrm{R}\mathrm{M}\mathrm{S}\mathrm{E}=\sqrt{\frac{\sum\:_{\mathrm{i}=1}^{\mathrm{m}}({\mathrm{Q}\mathrm{i}}_{\mathrm{c}\mathrm{a}\mathrm{l}}-{\mathrm{Q}\mathrm{i}}_{\mathrm{e}\mathrm{x}\mathrm{p}}{)}^{2}}{{\mathrm{m}}^{{\prime\:}}-\mathrm{p}}}$$

## Results and discussion

### Characterization of the adsorbent

**Fig. 3:**
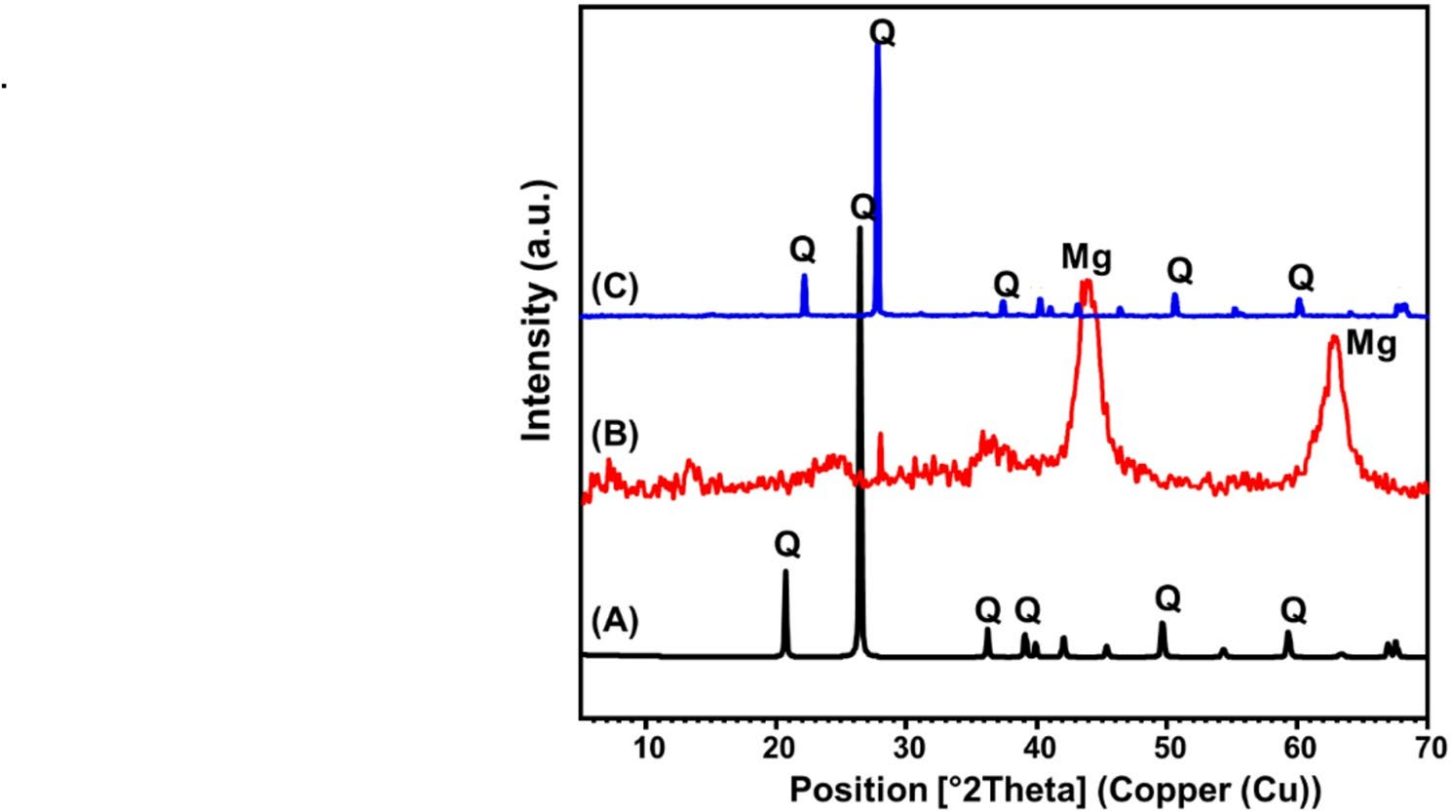
XRD patterns of raw quartz (**A**) [35], synthetic Mg/Al LDH (**B**) and synthetic Mg/GP particles (**C**)

The structural properties of the Mg/GP adsorbent and its precursor materials were investigated using X-ray diffraction (XRD) analysis (Fig. 3A–C). The raw quartz precursor exhibited a well-defined diffraction pattern, characteristic of highly crystalline quartz, with prominent peaks appearing at 2θ values of approximately 20.86°, 26.65°, 36.55°, 39.46°, and 50.18° (Fig. 3A), consistent with previously reported data [35, 38]. The XRD pattern of the calcined Mg/Al layered double hydroxide (LDH) revealed distinct peaks attributed to magnesium oxide (MgO) at 2θ ≈ 43° and 62.68°, corresponding to the standard diffraction pattern indexed in JCPDS card No. 01-077-2364 (Fig. 3B). The calculated crystallite size of MgO was approximately 4.8 nm, indicating nanoscale particle formation. Additionally, minor diffraction peaks corresponding to crystalline aluminum oxide (Al₂O₃) were detected and matched with JCPDS card No. 00-010-0173, confirming partial decomposition and crystallization of aluminum-containing phases upon calcination. Following the synthesis of the geopolymer (Mg/GP) through the incorporation of the Mg/Al–LDH and natural quartz in a strongly alkaline potassium hydroxide medium, the resulting XRD pattern retained the dominant features of quartz (Fig. 3C). However, notable reductions in peak intensity and slight shifts in peak positions were observed, suggesting partial dissolution of the quartz phase and its transformation into an amorphous or semi-amorphous geopolymer network. The absence of distinct MgO and Al₂O₃ reflections in the final product implies their successful integration into the geopolymer matrix, likely via chemical incorporation into the aluminosilicate framework or transformation into non-crystalline phases.

**Fig. 4:**
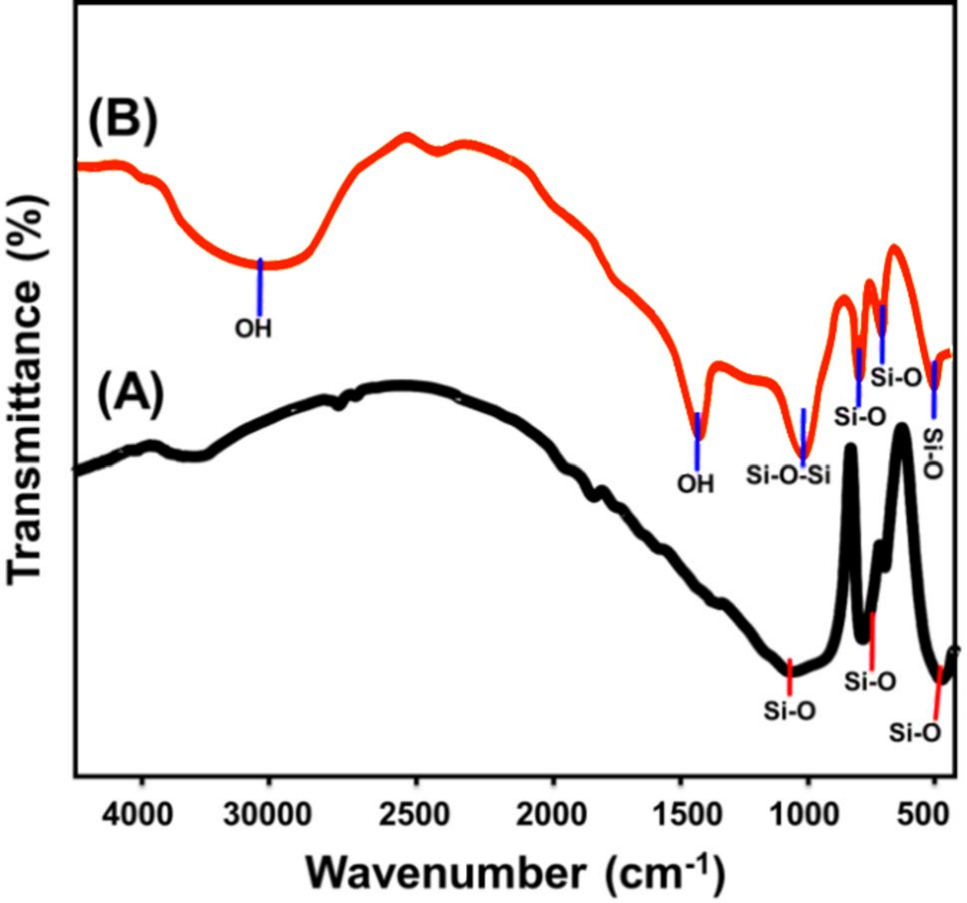
FT-IR spectra of raw quartz (**A**) [35] and synthetic Mg/GP particles (**B**)

Fourier-transform infrared (FT-IR) spectroscopy was employed to investigate the structural and chemical transformations occurring during the synthesis of the Mg/GP geopolymer. The FT-IR spectra of both natural quartz and the synthesized Mg/GP material provide clear evidence of successful geopolymerization (Fig. 4). In the spectrum of natural quartz (Fig. 4A), a prominent broad absorption band near 3500 cm⁻¹ is assigned to –OH stretching vibrations, typically associated with adsorbed water or trapped fluid inclusions within the quartz matrix [35, 50]. Additional characteristic bands were observed at approximately 473 cm⁻¹ (Si–O bending), 775 cm⁻¹ (symmetric Si–O stretching), and 1100 cm⁻¹ (asymmetric Si–O stretching), all indicative of highly ordered silica-based structures [51].

In contrast, the FT-IR spectrum of the synthesized Mg/GP geopolymer (Fig. 4B) revealed distinct spectral features reflecting successful chemical transformation and network formation. The broad absorption band at 3420 cm⁻¹ and the associated bending vibration at 1630 cm⁻¹ correspond to –OH groups, which are attributed to both physically adsorbed water and structural hydroxyls within the geopolymer matrix. These bands are consistent with the presence of amorphous aluminosilicate frameworks typically formed during polycondensation reactions in geopolymer systems [52]. Crucially, the band at 1028 cm⁻¹ is assigned to overlapping asymmetric stretching vibrations of Si–O–Si and T–O–Si (where T = Al or Mg), confirming the formation of a cross-linked three-dimensional aluminosilicate network. The presence of tetrahedral Si–O bending vibrations at 773 cm⁻¹ and 684.6 cm⁻¹ further suggests that remnants of the original quartz structure were preserved during synthesis, contributing to the final geopolymeric framework [53, 54].

These FT-IR observations confirm the effective condensation and interaction of SiO₄ and AlO₄ tetrahedral units, which form the structural basis of the geopolymer. Complementary energy-dispersive X-ray (EDX) analysis confirmed the presence of oxygen (O), silicon (Si), magnesium (Mg), aluminum (Al), and potassium (K) as the dominant elements in the Mg/GP composite. This elemental composition aligns well with the raw materials used and further validates the successful fabrication of the Mg/GP geopolymer.

**Fig. 5:**
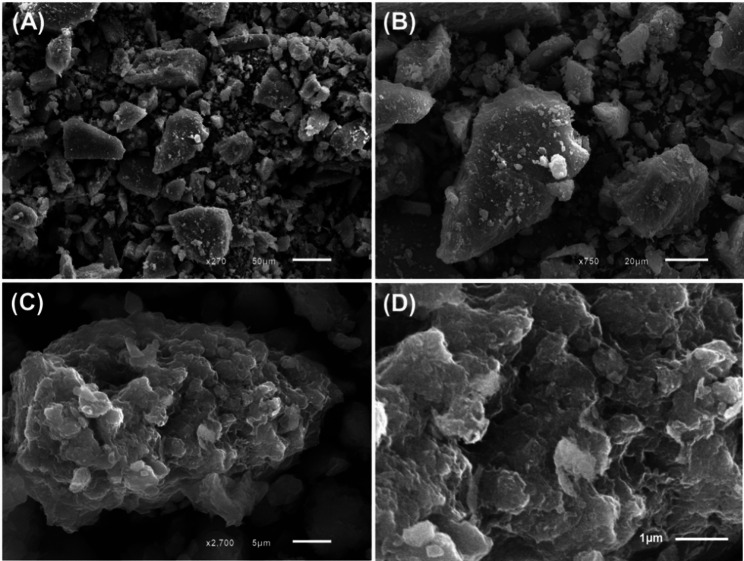
SEM images raw quartz (**A**,** B**) [35] and synthetic Mg/GP particles (**C**,** D**)

Significant morphological changes were observed during the transformation of the raw materials into geopolymer, as revealed by scanning electron microscopy (SEM) analysis (Fig. 5). The natural quartz precursor exhibited irregular, angular particles with relatively smooth surfaces and poorly defined edges—features commonly resulting from mechanical grinding processes (Fig. 5A, B). In contrast, the synthesized Mg/GP geopolymer displayed a markedly different morphology. The particles appeared as irregular, bulky agglomerates with a highly porous and rough surface texture, characteristic of geopolymeric materials (Fig. 5C, D). This transformation reflects successful polycondensation and structural reorganization during geopolymer synthesis.

The formation of porous, heterogeneous surfaces is strongly correlated with an increase in specific surface area, which was measured at 97.4 m²/g. The abundance of surface irregularities and interconnected pores significantly enhances the material’s adsorption capacity, supporting its effectiveness as an environmental adsorbent for water treatment applications.

.

### Environmental and health risk assessment of the groundwater

#### Statistical analysis of physicochemical parameters

**Fig. 6:**
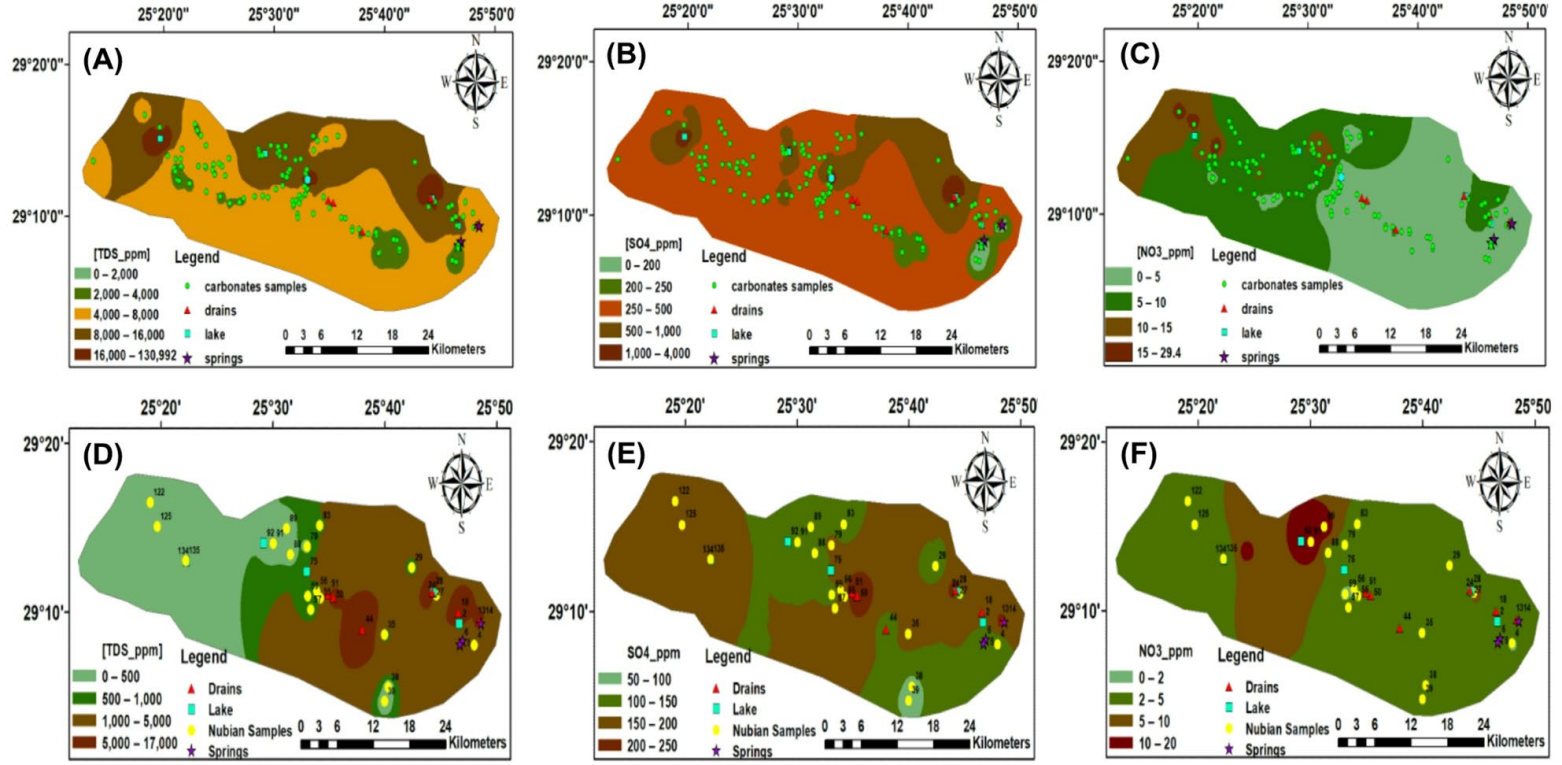
Distribution maps of physicochemical parameters (TCA (**A**–**C**) and NSSA (**D**–**F**)) [42, 44]

The evaluation of water resources in Egypt’s northwestern desert—specifically within the Siwa Oasis—was carried out by classifying collected water samples based on their physicochemical characteristics and ionic composition in accordance with established standards for drinking and irrigation (Table 1) [42, 44, 45]. The pH values of the sampled waters ranged from 6.4 to 8.9, reflecting neutral to slightly alkaline conditions, which are generally acceptable for both potable and agricultural applications. Electrical conductivity (EC) values exhibited significant variability across the different water sources, reflecting distinct salinity levels. Lake waters were identified as hypersaline, with EC values ranging from 23,250 to 188,800 µS/cm. Drainage waters demonstrated saline characteristics, with EC values between 8,390 and 20,490 µS/cm. In contrast, spring waters were categorized as brackish, with EC values ranging from 1840 to 11,790 µS/cm. The shallow aquifer system (Tertiary Carbonate Aquifer, TCA) displayed a wide EC range, spanning from 672 to 62,000 µS/cm, classifying it as brackish to saline depending on the location. Meanwhile, the deep aquifer system (Nubian Sandstone Aquifer System, NSSA) consistently exhibited freshwater quality, with EC values between 268 and 642 µS/cm, as illustrated in Fig. 6.

The Nubian Sandstone Aquifer System (NSSA) was identified as a highly suitable source for both drinking and irrigation purposes. Its ionic composition was predominantly characterized by the cation sequence Na⁺ > Ca²⁺ > Mg²⁺ > K⁺, with bicarbonate (HCO₃⁻) as the dominant anion, followed by chloride (Cl⁻), sulfate (SO₄²⁻), and nitrate (NO₃⁻) (Fig. 6). This distribution reflects natural geochemical processes, primarily driven by the dissolution of evaporite minerals and ion exchange interactions within the aquifer matrix. In contrast, the Tertiary Carbonate Aquifer (TCA) presented significant limitations for irrigation use. Elevated salinity levels and high concentrations of specific ions—particularly Na⁺, Mg²⁺, Cl⁻, and SO₄²⁻—were frequently observed, with many samples exceeding the Food and Agriculture Organization (FAO) recommended thresholds for agricultural water quality. The use of untreated TCA water for irrigation would likely pose substantial risks of soil salinization, necessitating advanced water treatment or management interventions to reduce its environmental impact and ensure sustainable agricultural use.

Specifically, ion concentrations in the Nubian Sandstone Aquifer System (NSSA) remained within acceptable limits for both drinking and irrigation. Measured values included sodium (18–280 mg/L), magnesium (2.3–56.5 mg/L), calcium (9.8–64.7 mg/L), and chloride (40.6–174 mg/L). In contrast, the Tertiary Carbonate Aquifer (TCA) exhibited elevated concentrations of several key ions. Magnesium, potassium, chloride, and sulfate exceeded FAO irrigation thresholds in 97.6%, 100%, 88%, and 2.3% of the analyzed samples, respectively—highlighting the limited suitability of this aquifer for direct agricultural use without prior treatment. Nitrate (NO₃⁻) concentrations were considerably higher in groundwater than in surface water. In the TCA, nitrate levels ranged from 1.4 to 29.4 mg/L, whereas in the NSSA, they varied from 1.4 to 14.2 mg/L. These elevated concentrations are attributed to both natural geogenic inputs and anthropogenic sources, including agricultural runoff and domestic wastewater infiltration. In many cases, nitrate concentrations exceeded permissible limits for drinking water, posing a significant public health risk, particularly for vulnerable groups such as children, due to the potential onset of methemoglobinemia and other nitrate-related disorders.

**Fig. 7:**
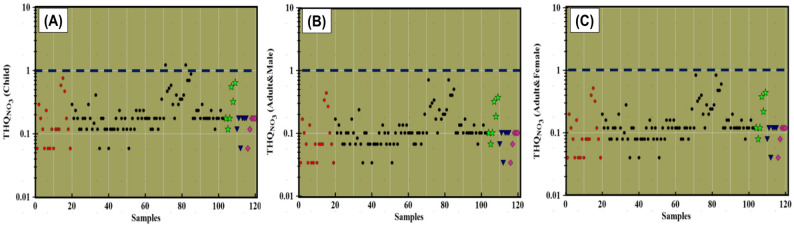
Scatter plots show the health risk indices regarding nitrates expossure in children, male, and female (**A**–**C**)

To visualize areas of concern, a spatial distribution map of the measured water quality parameters was developed using the kriging interpolation method, encompassing both aquifer systems and surface water bodies (Fig. 7). This geostatistical analysis effectively delineated high-risk zones within Siwa Oasis, providing critical insight into locations where environmental and health-related threats are most pronounced and where targeted intervention is urgently required. In conclusion, the Nubian Sandstone Aquifer System (NSSA) is identified as the most reliable and sustainable source of water for both drinking and irrigation purposes in the Siwa Oasis. Its low salinity and favorable ionic composition make it suitable for long-term use with minimal treatment. In contrast, the Tertiary Carbonate Aquifer (TCA) poses significant challenges due to high salinity levels and elevated concentrations of specific ions that exceed permissible limits for irrigation and, in some cases, for drinking. To mitigate these issues, targeted water treatment and soil management practices are essential. Recommended strategies include the cultivation of salt-tolerant crops and the establishment of regular monitoring programs, particularly for nitrate levels, to reduce associated health and environmental risks. These measures are crucial for ensuring the sustainable use of water resources and protecting both agricultural productivity and public health in the region.

#### Health risk regarding nitrate and sulfate exposure

A comprehensive health risk assessment was carried out for males, females, and children based on the U.S. Environmental Protection Agency (EPA) guidelines [47]. The assessment focused on evaluating non-carcinogenic risks associated with nitrate exposure through both oral ingestion and dermal contact, with the findings summarized in Table 1. Spatial distribution maps of the total hazard quotient (THQ) were generated using the kriging interpolation method to visualize spatial variations in health risk across the Siwa Oasis for each demographic group. The hazard quotient for dermal exposure (HQ _Dermal_) remained below 1 for all groups, indicating minimal risk via skin contact. However, oral ingestion emerged as the primary exposure pathway of concern. The hazard quotient for oral ingestion (HQ _Oral_) ranged from 0.03 to 0.71 (mean: 0.14) for males, 0.04 to 0.84 (mean: 0.16) for females, and 0.06 to 1.23 (mean: 0.24) for children, clearly indicating that children are more vulnerable to nitrate-related health effects. Notably, 1.66% of the analyzed water samples exceeded the EPA’s acceptable HQ threshold (> 1) for children, while no exceedances were observed for adult males or females. According to EPA standards, non-carcinogenic health risks are considered acceptable when THQ < 1, whereas values exceeding 1 may indicate potential adverse health effects. In this study, THQ values ranged from 0.059 to 1.23 for children, 0.034 to 0.71 for males, and 0.04 to 0.84 for females (Table 1). Out of 120 water samples analyzed, two samples—originating from the central and western zones of Siwa Oasis—exceeded the safe THQ threshold for children, identifying localized hotspots of concern. No such exceedances were found for adult populations, further emphasizing the need for age-specific monitoring and risk mitigation strategies, particularly for vulnerable groups like children.

These findings underscore the heightened susceptibility of children to nitrate-induced non-carcinogenic health effects within the Siwa Oasis. Elevated nitrate levels observed in the central and western regions of the oasis are likely associated with intensive agricultural practices and excessive groundwater extraction for irrigation. Such over-extraction can induce a cone of depression in the water table, facilitating the downward infiltration of nitrate-contaminated irrigation water back into the aquifer system. This mechanism aligns with previous research [55], which highlights children’s increased vulnerability due to their lower body weight, developing physiology, and behavioral factors that elevate exposure risk.

Spatial analysis of Total Hazard Quotient (THQ) values further revealed that males, females, and especially children residing in the central and western zones of the oasis face greater risk than those in the eastern areas. Notably, groundwater samples from the TCA aquifer, particularly samples S77 and S94, were deemed unsuitable for direct human consumption, owing to elevated THQ values exceeding the safe threshold. Chronic intake of nitrate-contaminated water has been linked to a range of adverse health effects, including hypertension, methemoglobinemia (blue baby syndrome), infant mortality, stomach cancer, thyroid dysfunction, goiter, migraines, skin rashes, cytogenetic damage, and congenital abnormalities [56, 57].

**Table 1:** Descriptive statistics of physical parameters, major ions, trace metals, and isotopic tracers in lakes, drains, springs, TCA, and NSSA supported with FAO and WHO standards

WHO	FAO	NSSA			TCA			Springs			Drains			Salt lakes			Parameter
Standard	Standard	Mean	Max.	Min.	Mean	Max.	Min.	Mean	Max.	Min.	Mean	Max.	Min.	Mean	Max.	Min.	
1000	2000	340	929	160	5002.31	38295.37	466.53	4996.66	8884.77	1463.05	9356.72	16125.75	5633.98	106137.94	153,589	57216.04	TDS
7.5	8.5	8.03	8.9	6.4	7.95	8.7	6.9	7.97	8.3	7.6	8.17	8.6	7.9	8.15	8.4	7.8	pH
1500	3000	396.29	642	268	7558.41	62000	672	6990	11790	1840	12430	20490	8390	120042.86	188,800	23250	EC
400	919	65	280	18	1052.65	10000	92	1044.38	1845	200	1913.13	3750	11.95	25058.57	39500	3455	Na
12	2	19	23	13	42.09	75.10	3.5	53.35	77.40	22.50	51.54	82.80	5.10	12.47	17.5	6.4	K
150	60	17.8	56.5	2.3	359.13	2524.28	9.04	339.79	583.05	167.23	566.01	888.26	266.67	6654.52	12216	1536.73	Mg
200	400	24.7	64.7	9.8	257.41	744	29.76	398.29	818.40	19.60	721.37	1568	104.16	1906.97	2508	1264.80	Ca
600	1036	93.9	174.0	40.6	2901.35	24360	174	2827.50	5220	725	5332.38	9744	3190	68937.14	94,250	48430	Cl
400	960	48.4	353.3	1.6	310.01	992.02	22.57	251.19	550	5.10	833.81	1850	300	3498.61	5348	2344	SO_4_
200	610	135.8	185.3	89.7	160.85	328.79	83.69	158.42	203.25	119.56	203.25	269.01	125.54	215.21	286	167	HCO_3_
45	…..	5.2	18.2	1.4	6.06	29.40	1.4	8.23	15.40	2.80	3.33	4.20	1.40	3.2	4.2	1.4	NO_3_
500	…..	130.11	265.06	63.7	2117.74	11858	186.20	2385.70	4185.00	931.00	4047.13	6370.00	1878.60	32026.29	55,664	9486	TH
	…..	39.5	43	35.5	24.9	37.6	17.7	21.75	25.1	17.5	20.3	21.1	18.8	19.93	27.1	13.5	T °C

The issue is particularly critical in regions where communities depend exclusively on untreated groundwater without routine water quality monitoring [57]. For instance, a study by Ravindra and Mor [58] in northern India found that 29 out of 80 groundwater samples posed significant non-carcinogenic health risks to children due to high nitrate levels. Similarly, He and Wu [59] reported that 87.6% of groundwater samples in China’s northern Shandong Peninsula were unsuitable for drinking based on nitrate concentrations, especially impacting infants and children through both oral ingestion and dermal contact. In line with these global findings, the present study highlights an urgent public health concern regarding nitrate contamination in groundwater within the Siwa Oasis. Immediate action by local and regional authorities is essential. This includes implementing nitrate reduction strategies, enhancing groundwater quality monitoring programs, promoting safe agricultural practices, and ensuring the provision of clean and sustainable drinking water for vulnerable populations, particularly children.

### Adsorption results

#### Effect of pH

**Fig. 8:**
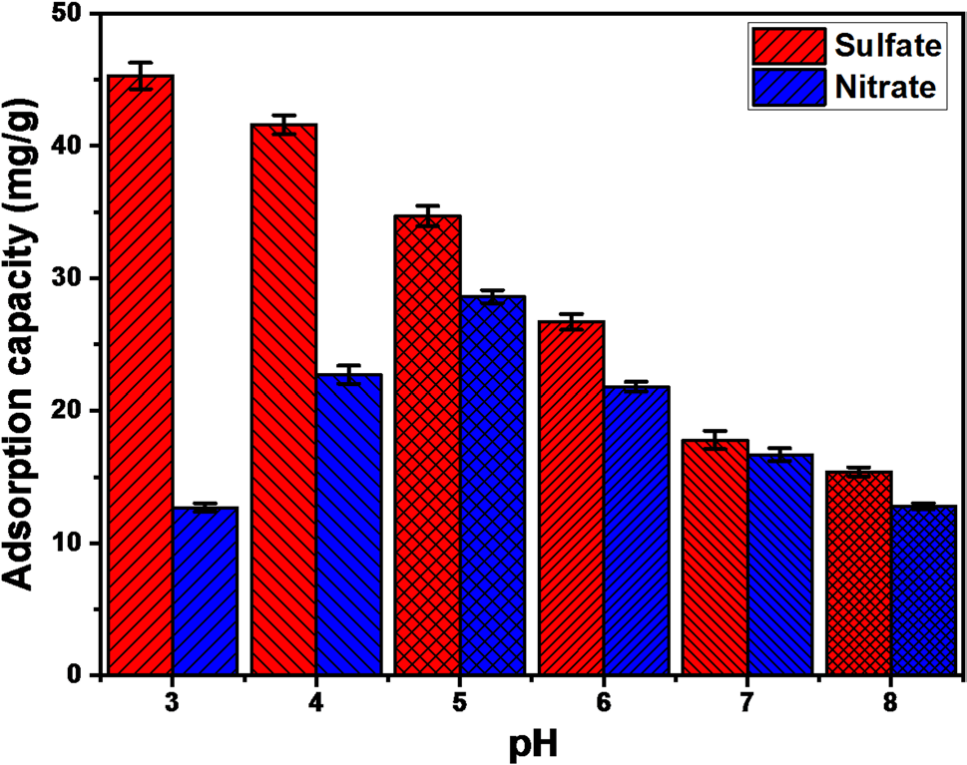
The experimental influence of pH on the uptake of SO₄²⁻ and NO₃⁻ ions by Mg/GP particles

The pH of the aqueous solution plays a critical role in modulating both the surface charge characteristics of the Mg/GP adsorbent and the ionization behavior of water-soluble species [60]. In this study, the effect of pH on adsorption performance was systematically investigated over a range of pH 3 to 8, while maintaining constant experimental conditions: contact time (60 min), temperature (293 K), solution volume (150 mL), initial concentrations of SO₄²⁻ and NO₃⁻ (50 mg/L each), and adsorbent dosage (0.3 g/L). For sulfate ions (SO₄²⁻), the adsorption capacity decreased markedly with increasing pH—from 45.4 mg/g at pH 3 to 15.3 mg/g at pH 8 (Fig. 8). This trend is attributed to the speciation behavior of sulfate and the pH-dependent surface charge of the Mg/GP material. According to prior studies, HSO₄⁻ predominates at pH < 2, while SO₄²⁻ becomes the dominant species above pH 2 [61, 62]. At higher pH levels, the deprotonation of surface hydroxyl groups on Mg/GP results in an increased negative surface charge, which electrostatically repels SO₄²⁻ ions, thereby reducing adsorption efficiency. In contrast, under acidic conditions, surface groups become protonated, generating a positive surface charge that enhances the electrostatic attraction of negatively charged sulfate species. Additionally, the high concentration of hydronium ions (H₃O⁺) at low pH values further facilitates adsorption through ion exchange and improved electrostatic interactions [63–65].

In contrast, the adsorption behavior of nitrate ions (NO₃⁻) exhibited a different trend. The adsorption capacity increased from 12.7 mg/g at pH 3 to a maximum of 28.6 mg/g at pH 5 (Fig. 8). At low pH (around pH 3), nitrate may be partially protonated to form nitric acid (HNO₃), reducing its electrostatic interaction with the positively charged Mg/GP surface [66]. As the pH increases, deprotonation of HNO₃ restores the negatively charged NO₃⁻ form, which interacts more effectively with the positively charged active sites on the adsorbent. This results in a peak adsorption capacity at pH 5 [66, 67]. However, beyond pH 5, the adsorption efficiency decreases due to the increased concentration of hydroxide ions (OH⁻). These negatively charged species compete with NO₃⁻ for available adsorption sites and contribute to electrostatic repulsion, thereby diminishing nitrate uptake [66, 68]. Overall, this study elucidates the ion-specific influence of pH on the adsorption performance of Mg/Al-based geopolymers, offering valuable insight into the design of pH-optimized water treatment systems tailored for the selective removal of sulfate and nitrate ions.

#### Contact time

A time-dependent adsorption study was conducted to evaluate the performance of Mg/GP in removing sulfate (SO₄²⁻) and nitrate (NO₃⁻) ions over a contact time range of 20 to 400 min. The experiments were performed under controlled conditions, with constant initial ion concentration (50 mg/L), temperature (20 °C), solution volume (150 mL), pH (3 for SO₄²⁻ and 5 for NO₃⁻), and adsorbent dosage (0.3 g/L). The objective was to determine the influence of contact time on adsorption efficiency and to identify the point of equilibrium.

**Fig. 9:**
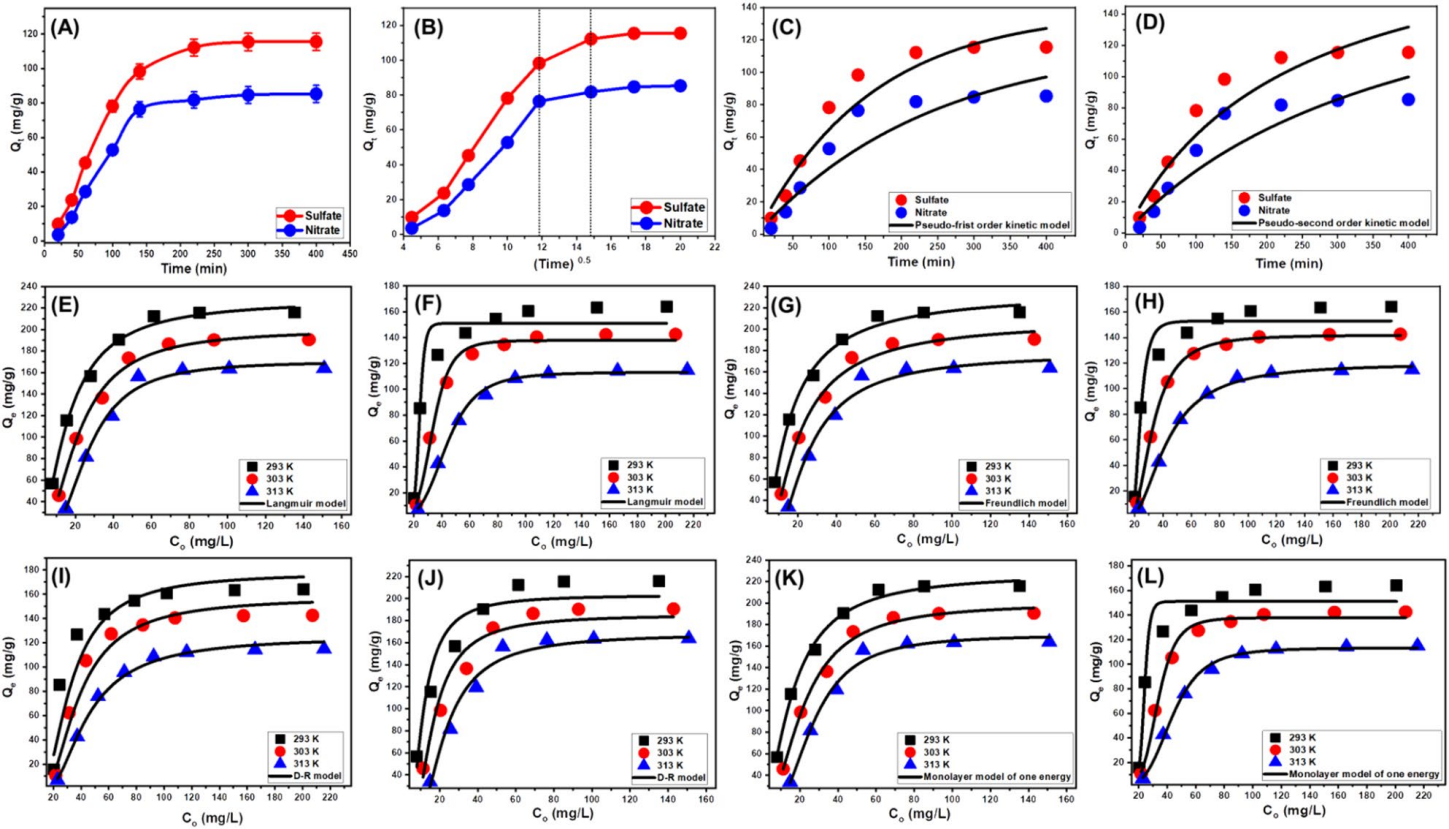
Shows the experimental influence of contact time on the uptake of SO₄²⁻ and NO₃⁻ ions by Mg/GP particles (**A**), the intra-particle diffusion curves (**B**), Pseudo-first-order kinetic modeling (**C**), Pseudo-second order kinetic modeling (**D**), classic Langmuir isotherm modeling (**E**,** F**), classic Freundlich isotherm modeling (**G**,** H**), classic D-R isotherm modeling (**I**,** J**), and advanced Monolayer isotherm model of one energy site (**K**,** L**)

The results revealed a pronounced increase in adsorption efficiency for both ions with increasing contact time, as evidenced by the uptake values and immobilized ion concentrations (Fig. 9A). A substantial enhancement in adsorption was observed up to approximately 300 min, beyond which the removal rates plateaued, indicating that adsorption equilibrium had been attained. At equilibrium (480 min), the adsorption capacities were 115.5 mg/g for SO₄²⁻ and 85.3 mg/g for NO₃⁻, demonstrating the high adsorption potential of Mg/GP under the tested conditions.

In the initial phase, a rapid increase in ion uptake was observed, attributed to the abundance of reactive and vacant adsorption sites on the surface of the geopolymer particles [69]. As time progressed, the number of available sites gradually diminished due to progressive ion sequestration, resulting in a decline in the adsorption rate. This decrease is primarily linked to the saturation of active functional groups, which limits the adsorbent’s capacity to capture additional ions. In the later stages, the adsorption curves leveled off, indicating that maximum adsorption capacity had been reached and no further ion binding occurred [14]. These findings confirm the high efficiency, stability, and time-dependent behavior of Mg/GP in the removal of SO₄²⁻ and NO₃⁻ ions. The results offer valuable insights into the kinetic performance of geopolymer-based adsorbents and support their application in long-duration water treatment processes.

#### Kinetic studies

#####  Intra-particle diffusion behavior

The intra-particle diffusion analysis offers valuable insight into the underlying adsorption mechanisms and binding behavior of sulfate (SO₄²⁻) and nitrate (NO₃⁻) ions onto the Mg/GP adsorbent. As shown in Fig. 9B, the adsorption curves reveal three distinct linear segments, each characterized by a different slope. This pattern indicates that the adsorption process proceeds through multiple sequential stages. Importantly, the non-zero intercepts of the curves—deviating from the origin—suggest that intra-particle diffusion is not the sole rate-controlling step, but rather part of a combined mechanism involving surface adsorption and diffusion-controlled processes [70].

The adsorption process can be broadly divided into three primary stages. The first stage involves rapid external surface adsorption, where SO₄²⁻ and NO₃⁻ ions interact immediately with available functional groups on the external surface of Mg/GP. This initial phase is dominant in the early moments of contact, driven by the high availability of unoccupied active sites, and accounts for the steep rise in adsorption capacity (Fig. 9B). As these surface sites become progressively saturated, the system transitions into the second stage, where intra-particle diffusion becomes the controlling mechanism. During this phase, ions begin to migrate into the internal porous network of the geopolymer structure. The continued uptake of SO₄²⁻ and NO₃⁻ in this stage underscores the importance of internal diffusion pathways in supporting prolonged adsorption and highlights the material’s structural capacity to accommodate ions beyond its surface [64, 71]. The third and final stage corresponds to the equilibrium phase, where all accessible adsorption sites—both external and internal—are occupied. At this point, the adsorption rate significantly decreases, and additional ion uptake is minimal. The stabilization of the process reflects a shift from rapid physical adsorption to weaker molecular interactions and interionic forces, which help maintain the adsorbed species on the surface [63, 72].

In summary, the adsorption of SO₄²⁻ and NO₃⁻ onto Mg/GP follows a multi-stage mechanism, encompassing both surface adsorption and intra-particle diffusion. This mechanistic understanding confirms the structural and functional efficiency of Mg/GP as a high-capacity adsorbent and reinforces its suitability for simultaneous multi-contaminant removal in water treatment applications. Additionally, the findings contribute to the broader understanding of geopolymer-based adsorbents by clearly distinguishing the roles of external binding and internal diffusion, thereby offering a valuable framework for optimizing future adsorbent design and performance.

##### Kinetic modeling

Modeling the kinetics of adsorption processes is essential for understanding the underlying mass transfer mechanisms, surface interactions, and the time-dependent behavior of adsorbate-adsorbent systems [73]. In this study, the removal kinetics of sulfate (SO₄²⁻) and nitrate (NO₃⁻) ions by the Mg/GP adsorbent were evaluated using two conventional models: the pseudo-first-order (PFO) and pseudo-second-order (PSO) kinetic models. The PFO model describes the rate of ion uptake relative to the availability of active binding sites, assuming a physical adsorption mechanism. In contrast, the PSO model considers adsorption as a chemisorption process involving valency forces or electron exchange between the adsorbent and adsorbate, often linked to the intrinsic physicochemical properties of the system.

Nonlinear regression analysis was applied to fit the experimental data to both models, and model performance was assessed based on the coefficient of determination (R²) and Chi-squared (χ²) error values (Table 2; Fig. 9C, D). The results indicated that the PFO model provided a superior fit for both anions, as evidenced by higher R² values and lower χ² errors compared to the PSO model. This suggests that the adsorption of SO₄²⁻ and NO₃⁻ onto Mg/GP is predominantly governed by physical interactions. The adsorption capacities predicted by the PFO model—138.2 mg/g for SO₄²⁻ and 119.7 mg/g for NO₃⁻—closely matched the experimental values obtained (Table 1), further validating the model’s reliability in describing the kinetics of the process.

The strong performance of the PFO model implies that physical adsorption mechanisms, such as van der Waals forces and electrostatic interactions, play a central role in the binding of sulfate and nitrate ions to the Mg/GP surface [74, 75]. Nevertheless, the PSO model also offered a reasonable approximation of the adsorption process, suggesting that chemical interactions—including hydrogen bonding and surface complexation—may contribute to ion uptake, though likely to a lesser extent [72, 75].

Furthermore, the adsorption process may involve layered deposition, where a physically adsorbed ion layer forms atop the initial chemically bound species, thereby enhancing overall uptake capacity [76]. This layered mechanism aligns with the observed kinetic behavior and supports the multi-mechanism nature of the adsorption process. Overall, the kinetic analysis confirms that Mg/GP exhibits fast and efficient adsorption of both SO₄²⁻ and NO₃⁻, primarily through physical binding, with potential contributions from secondary chemical interactions that enhance performance in longer contact durations.

#### Starting concentration

The effect of initial sulfate (SO₄²⁻) and nitrate (NO₃⁻) concentrations on adsorption performance was systematically evaluated using the Mg/GP adsorbent within a concentration range of 25–200 mg/L. To isolate the influence of concentration, all other experimental parameters were held constant: solution volume (150 mL), contact time (24 h), adsorbent dosage (0.3 g/L), pH (3 for SO₄²⁻ and 5 for NO₃⁻), and temperature (293–313 K). A positive correlation was observed between increasing initial anion concentrations and enhanced adsorption capacities (Fig. S1). This increase is attributed to the greater mass transfer driving force at higher concentrations, which facilitates diffusion of ions toward the available active sites on the Mg/GP surface [77]. Consequently, the adsorption efficiency improved significantly with increasing initial concentrations. However, this trend persisted only up to a threshold, beyond which further increases in ion concentration did not result in additional adsorption. This plateau effect is likely due to the saturation of available binding sites on the Mg/GP surface.

Determining the equilibrium behavior was essential to assess the maximum adsorption capacities under varying conditions. For SO₄²⁻, the maximum capacities were recorded as 215.8 mg/g at 293 K, 190.6 mg/g at 303 K, and 163.8 mg/g at 313 K. Similarly, for NO₃⁻, the maximum capacities were 164.1 mg/g at 293 K, 142.6 mg/g at 303 K, and 114.9 mg/g at 313 K (Fig. S1). The decline in adsorption capacity with increasing temperature for both anions suggests that the adsorption process is exothermic in nature. Elevated temperatures likely increase the thermal agitation of the adsorbate, thereby disrupting adsorbate–adsorbent interactions and reducing overall binding affinity. These findings underscore the importance of initial ion concentration and temperature as key parameters in optimizing the adsorption performance of Mg/GP. Understanding these relationships is critical for the design and operation of effective water treatment systems utilizing geopolymer-based adsorbents for multi-contaminant removal.

#### Classic isotherm models

Equilibrium evaluations were conducted to examine the distribution behavior of water-soluble contaminants in the presence of the Mg/GP adsorbent across a range of concentrations exceeding the equilibrium threshold. Conventional adsorption isotherm models are critical for understanding the nature of adsorbent–adsorbate interactions and optimizing the system’s performance. They provide insight into the selectivity of the adsorbent, the predicted ion uptake under equilibrium, and the maximum adsorption capacity of the system [76, 77]. In this study, the equilibrium adsorption characteristics of sulfate (SO₄²⁻) and nitrate (NO₃⁻) ions onto Mg/GP were assessed using three widely accepted isotherm models: Langmuir (Fig. 9E, F), Freundlich (Fig. 9G H), and Dubinin–Radushkevich (D–R) (Fig. 9I, J). Nonlinear regression was employed to fit experimental data to the theoretical models, and model performance was evaluated based on the correlation coefficient (R²) and Chi-squared (χ²) values.

The results showed that the Langmuir model best described the adsorption of SO₄²⁻ ions, suggesting a monolayer adsorption mechanism on a homogeneous surface with uniform binding sites. In contrast, the Freundlich model provided a better fit for NO₃⁻ adsorption, indicating that nitrate ions interact with heterogeneous surface sites and may form multilayer adsorption coverage on the Mg/GP surface. These findings imply that while sulfate adsorption occurs uniformly and predictably, nitrate adsorption is governed by more complex, site-dependent mechanisms. The favorability of adsorption for both anions was further confirmed by dimensionless separation factor (R_L_) values less than 1, indicating a favorable interaction between the adsorbent and the adsorbates [70]. The Langmuir-derived maximum adsorption capacities (Q_max_) for SO₄²⁻ were 227.6 mg/g at 293 K, 203.0 mg/g at 303 K, and 170.4 mg/g at 313 K. For NO₃⁻, the corresponding Q_max_ values were 171.1 mg/g, 147.9 mg/g, and 123.3 mg/g, respectively. In both cases, the adsorption capacity declined with increasing temperature, reinforcing the exothermic nature of the adsorption process and suggesting a reduced binding affinity at elevated thermal conditions.

The Dubinin–Radushkevich (D–R) isotherm model provided additional insights into the energetic characteristics of the adsorption process. Unlike the Langmuir and Freundlich models, the D–R model focuses on the mean adsorption energy (E), a parameter that helps distinguish between physical, chemical, or hybrid adsorption mechanisms [78]. In this study, the calculated E values for both sulfate (SO₄²⁻) and nitrate (NO₃⁻) fell within the range of 8–16 kJ/mol (Table 2), suggesting that the adsorption process involves a combination of physical and chemical interactions. This dual mechanism likely begins with weak physical forces, such as van der Waals interactions, which enable the initial attachment of ions to the adsorbent surface. As adsorption progresses, stronger chemical interactions—including hydrogen bonding and possible surface complexation—enhance the stability and overall adsorption capacity of Mg/GP. These findings align with earlier kinetic and equilibrium results, reinforcing that Mg/GP operates via a multi-mechanistic adsorption pathway.

**Table 2:** The estimated mathematical parameters of the studied kinetic, classic isotherm, and advanced equilibrium models

Material	Model		Parameter		Values		
Kinetic parameters							
Sulfate	Pseudo-First-order		***K***_***1***_ **(min**^**− 1**^**(**		0.0063		
			***Qe***_***(Cal)***_ **(mg/g)**		138.2		
			***R*** ^***2***^		0.94		
			***X*** ^***2***^		2.3		
	Pseudo-Second-order		***k***_***2***_ **(g mg**^**− 1**^ **min**^**− 1**^**)**		2.06 × 10^− 5^		
			***Qe***_***(Cal)***_ **(mg/g)**		208.3		
			***R*** ^***2***^		0.93		
			***X*** ^***2***^		2.87		
Nitrate	Pseudo-First-order		***K***_***1***_ **(min**^**− 1**^**(**		0.0042		
			***Qe***_***(Cal)***_ **(mg/g)**		119.7		
			***R*** ^***2***^		0.91		
			***X*** ^***2***^		4.07		
	Pseudo-Second-order		***k***_***2***_ **(g mg**^**− 1**^ **min**^**− 1**^**)**		1.22 × 10^− 5^		
			***Qe***_***(Cal)***_ **(mg/g)**		200.9		
			***R*** ^***2***^		0.89		
			***X*** ^***2***^		4.69		
Classic isotherm parameters							
					293 K	303 K	313 K
Sulfate		Langmuir		***Q***_***max***_ **(mg/g)**	227.6	200.1	170.4
				***b*** **(L/mg)**	0.012	0.0026	2.55 × 10^− 4^
				***R*** ^***2***^ ***X*** ^***2***^	0.99 0.20	0.99 0.22	0.99 0.26
				***RL***	0.30–0.76	0.6–0.93	0.95–0.99
		Freundlich		***k***_***F***_ **(mg/g)**	234.8	208.3	177.4
				***1/n***	0.68	0.62	0.52
				***R*** ^***2***^	0.98	0.97	0.98
				***X*** ^***2***^	1.22	1.32	0.92
		D-R model		***Q***_***m***_ **(mg/g)**	203.2	185.41	168.06
				***β*** **(mol**^**2**^**/kJ**^**2**^**)**	0.0078	0.0066	0.0063
				***E*** **(kJ/mol)**	8.1	8.7	8.9
				***R*** ^***2***^	0.94	0.96	0.98
				***X*** ^***2***^	2.13	1.38	0.7
Nitrate		Langmuir		***Q***_***max***_ **(mg/g)**	171.1	147.9	123.3
				***b*** **(L/mg)**	1.93 × 10^− 6^	5.33 × 10^− 9^	1.8 × 10^− 7^
				***R*** ^***2***^ ***X*** ^***2***^	0.97 1.49	0.99 0.32	0.99 0.13
				***RL***	0.99	0.99	0.99
		Freundlich		***k***_***F***_ **(mg/g)**	152.98	141.97	119.44
				***1/n***	0.14	0.29	0.39
				***R*** ^***2***^	0.98	0.99	0.99
				***X*** ^***2***^	1.19	0.014	0.05
		D-R model		***Q***_***m***_ **(mg/g)**	177.74	157.42	124.79
				***β*** **(mol**^**2**^**/kJ**^**2**^**)**	0.0058	0.0062	0.0069
				***E*** **(kJ/mol)**	9.2	8.9	8.5
				***R*** ^***2***^	0.91	0.97	0.99
				***X*** ^***2***^	5.27	1.57	0.16
Advanced isotherm parameters							
Sulfate	Monolayer model of one energy site			***n*** ***N***_***m***_ ***(mg/g)*** ***Q***_***sat***_ ***(mg/g)*** ***C***_***1/2***_ ***(mg/L)*** ***ΔE (KJ/mol)*** ***R*** ^***2***^ ***X*** ^***2***^	1.61 145.4 234.1 15.6 – 10.8 0.99 0.19	1.95 102.5 199.9 21.02 -16.8 0.99 0.22	2.53 67.2 170.1 26.2 – 20.4 0.99 0.28
Nitrate	Monolayer model of one energy site			***n*** ***Nm (mg/g)*** ***Q***_***sat***_ ***(mg/g)*** ***C1/2 (mg/L)*** ***ΔE (KJ/mol)*** ***R2*** ***X2***	8.83 18.8 166.1 23.9 – 15.1 0.97 1.49	5.42 27.4 148.5 33.4 – 15.9 0.99 0.32	4.11 29.5 121.2 43.3 – 16.9 0.99 0.13

#### Advanced isotherm models

The application of statistical physics methodologies to describe adsorption equilibria provides a comprehensive framework for analyzing the mechanistic and thermodynamic characteristics of adsorption processes. In this study, numerical simulations were utilized to evaluate the interactions between reactive functional groups—serving as adsorption receptors on the Mg/GP surface—and water-soluble contaminants, specifically sulfate (SO₄²⁻) and nitrate (NO₃⁻) ions. The applied mathematical models incorporated both steric and energetic parameters to yield reliable estimates of key adsorption descriptors. The steric parameters included Nm, representing the total number of adsorption sites occupied on the Mg/GP surface, and Q_sub_, the theoretical maximum adsorption capacity at saturation. Additionally, the average number of adsorbed ions per receptor site (n) was calculated to further elucidate the molecular-level adsorption mechanism. Energetic parameters evaluated included the internal energy (E_int_), entropy (Sa), adsorption energy (E), and enthalpy (G). These thermodynamic values provide insight into the stability and nature of ion–surface interactions under different conditions.

To ensure accurate parameter determination, the experimental equilibrium data were fitted using nonlinear regression and multivariable optimization algorithms, specifically the Levenberg–Marquardt iterative method. This approach allowed for precise modeling under the statistical assumptions of the chosen framework. The resulting fits demonstrated a strong agreement between the experimental and theoretical data, confirming that the adsorption process is best described by a monolayer adsorption mechanism involving single-site interactions (Fig. 9K, L; Table 2). The computed model parameters, summarized in Table 2, highlight the synergistic roles of steric constraints and energetic interactions in governing the adsorption process. The statistical physics approach not only enhances understanding of the binding mechanisms at a molecular level but also proves effective in predicting adsorption behavior for complex systems. These results further reinforce the potential of Mg/GP as a high-performance adsorbent in engineered water treatment systems targeting multicomponent contaminant removal.

##### Steric properties

######  Number of adsorbed ions per site (n)

The theoretical analysis of the n-value—representing the number of adsorbed ions per receptor site—offers crucial insights into the spatial orientation and interaction mechanisms of sulfate (SO₄²⁻) and nitrate (NO₃⁻) ions on the Mg/GP surface. This parameter provides valuable information on the configuration of adsorbed species, revealing whether interactions occur through horizontal or vertical/multilayered arrangements, which are particularly relevant in multi-ionic or multi-docking adsorption mechanisms [72, 79].

n-values < 1 typically indicate a horizontal molecular orientation, suggesting limited layering at each adsorption site. Such behavior is associated with multi-docking interactions, where several weak, non-covalent forces (e.g., van der Waals or dispersion forces) contribute to ion binding without multilayer formation. In contrast, n-values > 1 suggest vertical or non-parallel configurations, indicating that multiple ions are accommodated at a single adsorption site. These configurations are driven by stronger electrostatic interactions or hydrogen bonding, enabling multilayer adsorption and enhancing overall ion uptake capacity.

**Fig. 10:**
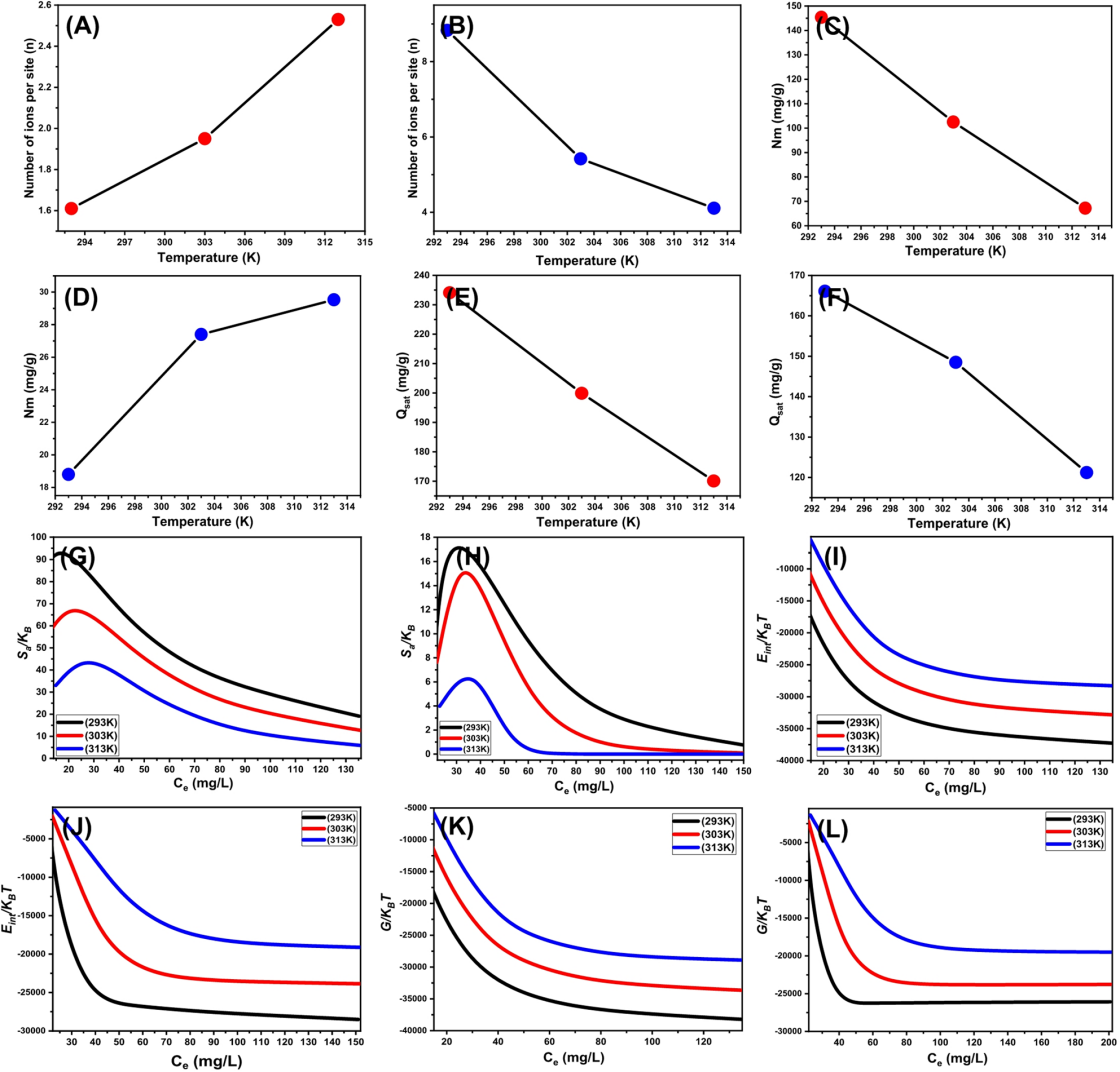
shows fitting changes in the number of adsorbed ions per site (SO₄²⁻ (**A**) and NO₃⁻ (**B**)), occupied site density (SO₄²⁻ (**C**) and NO₃⁻ (**D**)), saturation uptake capacity (SO₄²⁻ (**E**) and NO₃⁻ (**F**)), entropy (SO₄²⁻ (**G**) and NO₃⁻ (**H**)), internal energy (SO₄²⁻ (**I**) and NO₃⁻ (**J**)), and free enthalpy (SO₄²⁻ (**K**) and NO₃⁻ (**L**))

In this study, the calculated n-values ranged from 1.6 to 2.53 for SO₄²⁻ (Fig. 10A) and from 4.11 to 8.8 for NO₃⁻ (Fig. 10B). These values confirm the dominance of multi-ionic adsorption mechanisms for both anions. Specifically, individual receptor sites on the Mg/GP surface were capable of binding up to 3 SO₄²⁻ ions and up to 9 NO₃⁻ ions, arranged predominantly in vertical or stacked orientations. The significantly higher n-values for NO₃⁻ may be attributed to its smaller ionic radius, higher diffusivity, and lower charge density, which collectively enhance its ability to aggregate at adsorption sites.

A notable temperature-dependent behavior was observed. For SO₄²⁻, n-values increased with rising temperature, suggesting enhanced aggregation due to thermal activation. This trend implies that higher temperatures reduce the hydration shell of sulfate ions, allowing them to approach the adsorbent surface more closely and interact more effectively, thus facilitating multi-ion binding. Conversely, for NO₃⁻, n-values decreased with increasing temperature, indicating a reduction in aggregation behavior. This may result from weakened electrostatic interactions or increased desorption, as thermal energy disrupts the stabilizing forces that promote multilayer formation.

These contrasting trends highlight the ion-specific adsorption mechanisms of SO₄²⁻ and NO₃⁻ and underscore the complex role of ionic size, hydration energy, and temperature in influencing molecular arrangement and adsorption site saturation. Collectively, these findings contribute a deeper mechanistic understanding of how Mg/GP accommodates multiple ions per site and how thermal conditions modulate its adsorption behavior. This knowledge is instrumental in guiding the design of thermally optimized geopolymer-based adsorbents for selective and efficient multi-ion removal in advanced water treatment applications.

###### Density of the active sites (Nm)

The evaluation of functional uptake site densities (Nm) for sulfate (SO₄²⁻) and nitrate (NO₃⁻) ions provides a quantitative assessment of the total number of adsorption sites occupied on the Mg/GP surface under varying thermal conditions (Fig. 10C, D). For SO₄²⁻, the Nm values decreased significantly with increasing temperature, measured at 145.4 mg/g at 293 K, 102.5 mg/g at 303 K, and 67.2 mg/g at 313 K (Fig. 10C). This trend indicates a temperature-induced reduction in adsorption site saturation, suggesting that higher temperatures negatively impact the activation, retention, and stability of functional binding sites for sulfate ions.

These results align with prior findings which show that elevated temperatures enhance desorption and ion mobility, thereby lowering the occupancy of adsorption sites under thermal stress [1, 80]. Additionally, increased temperature can disrupt hydrogen bonding and electrostatic interactions, especially for multivalent ions such as SO₄²⁻, resulting in shorter residence times and diminished adsorption efficiency [81, 82]. In contrast, the Nm values for NO₃⁻ ions exhibited a different trend, increasing modestly with temperature: 18.8 mg/g at 293 K, 27.4 mg/g at 303 K, and 29.5 mg/g at 313 K (Fig. 10D). This suggests a slight improvement in adsorption site accessibility or activation with rising temperature. This behavior may be attributed to the lower charge density and weaker hydration shell of NO₃⁻ compared to SO₄²⁻, which allows for easier penetration and interaction with active sites on the Mg/GP surface.

These contrasting temperature-dependent behaviors underscore the influence of ionic properties, particularly valence charge, size, hydration energy, and mobility, on adsorption mechanisms. The stronger electrostatic attraction between divalent SO₄²⁻ and the adsorbent surface enhances its binding under ambient conditions but also makes it more sensitive to thermal disruption. Conversely, the monovalent NO₃⁻ ion, while interacting more weakly, exhibits greater thermal resilience due to its enhanced diffusivity and lower hydration energy [83–85]. Overall, these findings provide important insights into the ion-specific adsorption dynamics of Mg/GP under varying temperatures. They highlight that SO₄²⁻ adsorption is largely governed by binding site saturation and thermal desorption, whereas NO₃⁻ uptake is influenced by ionic mobility and aggregation behavior. This understanding is essential for designing thermally optimized geopolymer-based adsorbents for selective and efficient contaminant removal in dynamic environmental conditions.

###### Adsorption capacity at the saturation state of (*Q*_*sat*_)

The saturated adsorption capacity of Mg/GP (Q_sat_) represents the maximum ion removal efficiency and reflects the material’s overall binding tolerance and affinity toward water-soluble contaminants. This capacity is governed primarily by two key parameters: the total number of occupied adsorption sites (N_m_) and the average number of ions adsorbed per site (n). For sulfate ions (SO₄²⁻), Q_sat_ values were found to be 234.1 mg/g at 293 K, 199.9 mg/g at 303 K, and 170.1 mg/g at 313 K (Fig. 10E). In comparison, the corresponding Q_sat_ values for nitrate ions (NO₃⁻) were 166.1 mg/g at 293 K, 148.5 mg/g at 303 K, and 121.2 mg/g at 313 K (Fig. 10F). The consistently higher Q_sat_ values observed for sulfate across all temperatures highlight the stronger electrostatic interactions between the divalent SO₄²⁻ ions and the functional sites on the Mg/GP surface. This behavior can be attributed to the greater charge density of SO₄²⁻, which enhances its binding affinity, and its smaller hydration radius compared to NO₃⁻, allowing more efficient diffusion and penetration into the porous structure of the adsorbent.

These findings are consistent with earlier studies that reported preferential sulfate adsorption over nitrate on porous adsorbents. Furthermore, the observed temperature-dependent decline in Q_sat_ values for both anions supports the exothermic nature of the adsorption process. Elevated thermal energy likely increases molecular motion and collision frequency, weakening the adsorbate–adsorbent interactions and reducing the overall adsorption efficiency. Notably, the Q_sat_ trend for sulfate closely followed the changes in N_m_, suggesting that its adsorption is primarily governed by the total density of available binding sites. In contrast, nitrate’s Q_sat_ values corresponded more strongly with variations in the n parameter, indicating that nitrate adsorption is largely influenced by aggregation behavior and the ability of each receptor site to accommodate multiple ions.

These contrasting behaviors reveal distinct adsorption mechanisms for the two anions. Sulfate removal appears to depend more on the extent of surface site availability and binding saturation, while nitrate adsorption is driven by ion-specific interactions and per-site multi-ionic accumulation. This mechanistic differentiation provides important insight into how Mg/GP functions under varying thermal and chemical conditions and supports its use as a thermally responsive, ion-selective adsorbent in advanced water treatment applications.

#####  Energetic properties

###### Adsorption energy

The analysis of adsorption energy changes (ΔE) during the removal of sulfate (SO₄²⁻) and nitrate (NO₃⁻) ions provides critical insight into the nature of the adsorption mechanisms, allowing differentiation between physical and chemical interactions. This understanding is essential for optimizing adsorbent design, regeneration strategies, and overall system performance. Adsorption mechanisms are typically classified based on ΔE thresholds: physisorption occurs when ΔE is below 40 kJ/mol, whereas chemisorption is associated with values above 80 kJ/mol.

Within the physisorption range, various interaction types contribute to ion binding, including hydrogen bonding (ΔE < 30 kJ/mol), dipole–dipole interactions (ΔE = 2–29 kJ/mol), van der Waals forces (ΔE = 4–10 kJ/mol), electrostatic attraction (ΔE = 2–50 kJ/mol), and hydrophobic interactions (ΔE ≈ 5 kJ/mol). These energy ranges provide a valuable reference framework for interpreting the dominant forces driving ion adsorption onto Mg/GP.

In this study, the adsorption energy (ΔE) was calculated using the thermodynamic Eq. (10):

10 $$\:\varDelta\:E=RT\:ln\left(\frac{S}{C}\right)$$

where *R* is the universal gas constant (0.008314 kJ/mol·K), *T* is the system temperature, *S* is the ion solubility, and *C* is the concentration at the half-saturation level of the Mg/GP adsorbent.

Based on this formulation, the calculated ΔE values were approximately − 20 kJ/mol for SO₄²⁻ and − 17 kJ/mol for NO₃⁻ (Table 2), clearly falling within the physisorption range. These values indicate that the adsorption of both ions is primarily governed by hydrogen bonding, electrostatic attraction, and dipole–dipole interactions. Hydrogen bonds likely form between surface hydroxyl groups on the Mg/GP adsorbent and the electronegative oxygen atoms in the sulfate and nitrate anions. The specific functional groups and molecular structures of both the adsorbent and anions create favorable conditions for such interactions [86, 87]. Furthermore, the negative sign of ΔE confirms that the adsorption process is exothermic and spontaneous, indicating energetically favorable binding events. The role of electrostatic forces is particularly significant, given the attraction between the negatively charged anions and the positively charged functional groups present on the Mg/GP surface, further stabilizing the adsorbed species.

Importantly, the dominance of physisorption in the interaction between Mg/GP and both SO₄²⁻ and NO₃⁻ has practical implications. Due to the relatively low binding energies, adsorbed ions can be efficiently desorbed, supporting the regeneration and reuse of the Mg/GP material. This reversibility enhances the economic feasibility and operational sustainability of Mg/GP in large-scale environmental applications, including wastewater treatment and remediation of nitrate- and sulfate-contaminated water sources.

######  Entropy

The entropy (Sa) associated with the adsorption of sulfate (SO₄²⁻) and nitrate (NO₃⁻) ions onto Mg/GP offers valuable insight into the degree of molecular ordering or disorder at the adsorbent–adsorbate interface, particularly under varying ion concentrations and temperature conditions. Entropy behavior was evaluated using Eq. (11), which integrates key steric and kinetic parameters, including the site density (N_m_), the number of ions adsorbed per site (n), and the ion concentration at half-saturation (C_1/2_):

11 $$\:\frac{{S}_{a}}{{K}_{B}}=Nm\left\{{ln}\left(1+{\left(\frac{C}{{C}_{\frac{1}{2}}}\right)}^{n}\right)-n{\left(\frac{C}{{C}_{\frac{1}{2}}}\right)}^{n}\:\frac{ln\left(\frac{C}{{C}_{\frac{1}{2}}}\right)}{1+{\left(\frac{C}{{C}_{\frac{1}{2}}}\right)}^{n\:}}\:\:\right\}$$

This model allows for a quantitative assessment of configurational entropy changes during ion binding.

Analysis of the entropy profiles revealed a notable reduction in Sa during SO₄²⁻ adsorption, particularly at higher ion concentrations (Fig. 10G and H). This entropy decrease indicates increasing surface ordering, as sulfate ions progressively occupy available binding sites on the Mg/GP surface. The observed trend suggests improved sulfate docking efficiency, even at relatively low initial concentrations, which reflects a strong affinity for unoccupied sites and rapid surface coverage [80, 81]. The maximum entropy values for SO₄²⁻ adsorption occurred at equilibrium concentrations of 15.3 mg/L (293 K), 20.4 mg/L (303 K), and 25.5 mg/L (313 K) (Fig. 10G), while the corresponding values for NO₃⁻ were 24.4 mg/L, 31.3 mg/L, and 37.2 mg/L at the same respective temperatures (Fig. 10H). These values closely approximate the C_1/2_ concentrations, indicating that entropy reaches its peak at half-saturation—beyond which further adsorption results in diminished surface freedom due to site saturation. The continued decline in entropy at higher ion concentrations implies a reduction in the number of accessible adsorption sites, corresponding to decreased ion mobility, constrained diffusion, and increasing structural rigidity at the adsorbent interface. This behavior reflects a progressive loss of configurational disorder, marking the transition from a dynamic, flexible adsorption regime to one dominated by restricted surface reorganization during the late stages of ion uptake [86].

This study represents the first entropy-based evaluation of SO₄²⁻ and NO₃⁻ adsorption onto a Mg/Al-based geopolymer. The results provide a novel thermodynamic perspective, revealing how ion concentration and temperature modulate surface ordering, molecular confinement, and adsorption site availability. These insights are essential for understanding the limits of adsorption performance and for designing efficient, entropy-informed adsorbent systems capable of handling multi-ionic contaminants under diverse environmental conditions.

###### Internal energy and free enthalpy

This study evaluated the internal energy (E_int_) and free enthalpy (G) associated with the adsorption of sulfate (SO₄²⁻) and nitrate (NO₃⁻) ions onto Mg/GP, with particular focus on the effects of ion concentration and temperature variation. These thermodynamic parameters were calculated using Eqs. (12) and (13), which incorporate key adsorption variables, including the adsorption site density (N_m_), average number of ions adsorbed per site (n), half-saturation concentration (C_1/2_), and the translational partition function (Zv) [88]:

12 $$\:\frac{{E}_{int}}{{K}_{B}T\:}=\:n\:{N}_{m}\left[\left(\:\frac{{\left(\frac{C}{{C}_{1/2}}\right)}^{n}\:ln\left(\frac{C}{{Z}_{v}}\right)}{1+{\left(\frac{C}{{C}_{1/2}}\right)}^{n}}\right)-\left(\:\frac{n\mathrm{ln}\left(\frac{C}{{C}_{1/2}}\right)\:{\left(\frac{C}{{C}_{1/2}}\right)}^{n}}{1+{\left(\frac{C}{{C}_{1/2}}\right)}^{n}}\right)\right]$$

13 $$\:\frac{G}{{K}_{B}T\:}=\:n\:{N}_{m}\frac{\mathrm{ln}\left(\frac{C}{{Z}_{v}}\right)}{1+{\left(\frac{{C}_{1/2}}{C}\right)}^{n}}$$

The calculated E_int_ values for both ions were consistently negative across all tested temperatures, confirming that the adsorption of SO₄²⁻ and NO₃⁻ onto Mg/GP is both spontaneous and exothermic. A progressive decline in E_int_ was observed as the temperature increased from 293 K to 313 K (Fig. 10I, J), indicating a reduction in adsorption efficiency with increasing thermal energy. This temperature-dependent decrease in internal energy suggests that elevated temperatures weaken the interactions between the adsorbate and the active sites on the Mg/GP surface. imilarly, the free enthalpy (G) exhibited a parallel trend, with a reduction in magnitude as temperature rose (Fig. 10K, L). This behavior reinforces the conclusion that the thermodynamic favorability of adsorption diminishes at higher temperatures. The consistent decline in both E_int_ and G with increasing temperature reflects the thermal sensitivity of the adsorption system and confirms the exothermic character of the interaction.

Together, these findings validate that the adsorption of both SO₄²⁻ and NO₃⁻ is governed by energetically favorable mechanisms that are highly reversible and temperature-sensitive. Understanding these trends is essential for optimizing the practical application of Mg/GP in temperature-variable water treatment systems, where thermal fluctuations may influence adsorption efficiency and system design.

#### Recyclability

The recyclability of the synthesized Mg/GP adsorbent is a critical parameter for assessing its commercial viability and long-term practicality in water treatment applications. To evaluate this property, a regeneration protocol was employed, involving repeated rinsing with distilled water, with each cycle lasting 15 min. Following rinsing, the Mg/GP particles were dried at 60 °C for 12 h in an electric oven to prepare them for subsequent adsorption cycles targeting sulfate (SO₄²⁻) and nitrate (NO₃⁻) ions. All experimental conditions influencing adsorption performance were held constant across the five regeneration cycles: solution volume (150 mL), contact time (24 h), adsorbent dosage (0.3 g/L), pH (3 for SO₄²⁻ and 5 for NO₃⁻), initial ion concentration (200 mg/L), and temperature (293 K). This standardized approach allowed for a reliable assessment of reusability and performance degradation over multiple cycles.

The results demonstrated that Mg/GP retained excellent regeneration efficiency and structural stability over five successive adsorption–desorption cycles. For SO₄²⁻, the adsorption capacity remained high at 210.3 mg/g after two cycles, decreased slightly to 194.5 mg/g after three cycles, and retained a respectable 178.7 mg/g after five cycles (Fig. S2). Similarly, for NO₃⁻, Mg/GP maintained adsorption capacities of over 160 mg/g after two cycles, 148.6 mg/g after three cycles, and 130.2 mg/g after five cycles (Fig. S2). These findings confirm the durability, reusability, and consistent performance of Mg/GP, even after repeated regeneration. The material exhibits strong resistance to degradation, supporting its use in sustainable and cost-effective water treatment systems. The ability to maintain high removal efficiencies over multiple cycles highlights Mg/GP’s potential for real-world application, particularly in large-scale or continuous-flow environmental remediation technologies where adsorbent longevity and regeneration are crucial to operational success.

####  Comparative study

The comparative analysis presented in Table S2 evaluates the adsorption capacities of various reported materials for sulfate and nitrate removal, emphasizing the superior performance of the synthesized magnesium-based geopolymer (Mg/GP) relative to conventional adsorbents. Mg/GP exhibited the highest adsorption capacities among the reviewed materials, achieving 234.1 mg/g for sulfate and 166.1 mg/g for nitrate, thereby outperforming a wide range of adsorbents commonly studied in the literature. For sulfate adsorption, Mg/GP demonstrated a significantly higher capacity compared to traditional materials such as microfibrillated cellulose (7.35 mg/g), Mg–Fe calcined layered double hydroxide (68.7 mg/g), graphene oxide (26.83 mg/g), and iron hydroxide (48.03 mg/g). Even when compared with high-performance adsorbents like Mg–Al LDH (135.1 mg/g) and GAC/Mg–Al LDO composites (143.5 mg/g), Mg/GP showed a marked improvement in removal efficiency. Similarly, in the context of nitrate removal, Mg/GP outperformed notable adsorbents such as Al-modified biochar (89.5 mg/g), graphene (89.9 mg/g), and biochar-supported polyaniline (72 mg/g).

This outstanding adsorption performance can be attributed to the high surface area, abundant active binding sites, and favorable ion-exchange capacity of Mg/GP. The material’s unique structure, synthesized from a combination of Mg/Al LDH and natural quartz, offers enhanced porosity, mechanical integrity, and functional group availability. Unlike synthetic silica, the use of natural quartz not only contributes to the material’s structural performance but also significantly reduces production costs, making the process more economically viable. Furthermore, the presence of Mg²⁺, K⁺, and Na⁺ cations from the LDH phase improves ion-exchange kinetics, thus contributing to the elevated adsorption capacities for both anions. Given its high adsorption efficiency, cost-effectiveness, and scalability, Mg/GP emerges as a promising adsorbent for large-scale water treatment applications. Its ability to remove sulfate and nitrate to levels compliant with WHO drinking water standards highlights its relevance for real-world deployment in both developed and resource-limited settings. The material’s regeneration potential, coupled with the availability of low-cost precursors, positions Mg/GP as a sustainable and innovative solution for addressing global water contamination challenges.

#### Realistic study

To assess its practical applicability, the synthesized Mg/GP composite was evaluated for its ability to remove sulfate (SO₄²⁻) and nitrate (NO₃⁻) ions from real water matrices, including a representative groundwater sample collected from multiple wells in the Siwa Oasis and from seawater under controlled laboratory conditions. The experiments were conducted using varying adsorbent dosages (0.4–2 g/L), with a fixed solution volume of 1000 mL, contact time of 24 h, and a constant room temperature of 25 °C. The initial average concentrations in the groundwater samples were 456.2 mg/L for SO₄²⁻ and 10.4 mg/L for NO₃⁻. The results demonstrated a strong and dose-dependent adsorption capability of Mg/GP for both target ions. At a dosage of 2 g/L, the concentration of SO₄²⁻ was reduced by approximately 72.4%, from 456.2 mg/L to 125.7 mg/L, which falls well within the WHO recommended limit of < 250 mg/L (Table 3). This corresponds to a sulfate adsorption capacity of up to 330.5 mg, which could potentially be recovered via physical or chemical desorption methods or reused as a nutrient source, particularly in fertilizer applications.

Similarly, the application of 1.6 g/L of Mg/GP achieved an impressive 94.2% removal efficiency for NO₃⁻, reducing its concentration from 10.4 mg/L to 0.6 mg/L. This corresponds to a nitrate adsorption capacity of approximately 9.8 mg. Beyond environmental remediation, this level of nitrate capture also holds commercial potential, as recovered nitrate may be reclaimed for agricultural use as a nitrogen-rich supplement. These results confirm the exceptional effectiveness, selectivity, and reusability of Mg/GP in treating complex aqueous environments, even in the presence of competing ions. The material demonstrated high performance in both freshwater and saline systems, further underscoring its versatility. The combination of robust adsorption capacity and recoverable ion content highlights Mg/GP as a promising candidate for real-world water treatment and resource recovery applications. Its dual benefits—pollutant removal and nutrient reclamation—offer a compelling pathway for sustainable environmental management and circular economy integration in water treatment strategies.

**Table 3:** Realistic adsorption of SO₄²⁻ and NO₃⁻ from groundwater using Mg/GP structure

Dose (g/L)	Remaining (mg/L)	Removal (%)	Capacity (mg/g)
SO₄²⁻			
Control	456.2 mg/L	–	–
0.4 g/L	408.7 mg/L	10.4	118.7 mg/g
0.8 g/L	340.6 mg/L	25.3	144.5 mg/g
1.2 g/L	268.8 mg/L	41.1	156.2 mg/g
1.6 g/L	200.3 mg/L	56.1	159.9 mg/g
2 g/L	125.7 mg/L	72.4	165.2 mg/g
NO₃⁻			
Control	10.4 mg/L	–	–
0.4 g/L	6.7 mg/L	35.6	9.3 mg/g
0.8 g/L	3.4 mg/L	67.3	8.7 mg/g
1.2 g/L	1.6 mg/L	84.6	7.3 mg/g
1.6 g/L	0.6 mg/L	94.2	6.1 mg/g

## Conclusions

This study investigated the synthesis and performance of Mg/Al LDH and quartz-based geopolymer (Mg/GP) as an adsorbent for the removal of sulfate (SO₄²⁻) and nitrate (NO₃⁻) ions from aqueous systems. The results demonstrate that the synthesized material exhibits appreciable adsorption capacity, with maximum uptake values of 234.1 mg/g for sulfate and 166.1 mg/g for nitrate under optimized laboratory conditions. The adsorption behavior was satisfactorily described using equilibrium and kinetic models, and the statistical physics-based analysis provided insight into the dominant adsorption mechanisms, indicating that physical interactions such as electrostatic attraction and hydrogen bonding play a major role. The evaluation of groundwater samples from the Siwa Oasis further highlighted the environmental relevance of this work, as elevated concentrations of nitrate and sulfate were identified in several locations. Application of the Mg/GP material to real water samples resulted in measurable reductions in both contaminants, demonstrating its practical applicability under realistic conditions.

However, it should be noted that these results were obtained under controlled experimental settings, and further investigation is required to assess long-term performance, regeneration efficiency, and behavior under variable field conditions. Overall, the findings suggest that Mg/GP is a promising low-cost and environmentally compatible adsorbent for the mitigation of nitrate and sulfate contamination. Nevertheless, additional studies, including pilot-scale testing and long-term stability assessments, are recommended before large-scale implementation. This work contributes to the growing body of research aimed at developing sustainable materials for water treatment while acknowledging the limitations and future research needs inherent to laboratory-scale investigations.

## Recommendations

Future work will focus on fine-tuning the synthesis parameters to further enhance the porosity and active site density of Mg/GP, thereby increasing its adsorption efficiency. The modification of the geopolymer to target additional contaminants—such as heavy metals or organic pollutants—will be explored, broadening its applicability in diverse environmental remediation scenarios.

## Supplementary Information

Below is the link to the electronic supplementary material.

Supplementary Material 1.

## Data Availability

The data will be available up on request to corresponding author.
