# Supplementary material for: Health risk assessment and removal of nitrate and sulfate ions using Mg/Al quartz-based geopolymer: a case study from Siwa Oasis
Source: BMC Chem. 2026 Jan 16;20(1):22. doi: 10.1186/s13065-026-01721-4 (PMC12892519; doi:10.1186/s13065-026-01721-4)
Supplement: Supplementary file 1 — Supplementary Material 1. [file 13065_2026_1721_MOESM1_ESM.docx]

**Fig.S1.** shows the experimental influence of starting concentrations on the uptake of SO₄²⁻ (A) and NO₃⁻ (B) ions by Mg/GP particles (A)

**Table S1.** Nonlinear equations of kinetic, classic isotherm, and advanced isotherm models

| Kinetic models | | |
| --- | --- | --- |
| Model | **Equation** | **Parameters** |
| Pseudo-first-order | $Q_{t}=Q_{e} (1-e^{{-k}_{1}.t})$ | Q_t_ (mg/g) is the adsorbed ions at time (t), and K_1_ is the rate constant of the first-order adsorption (1/min) |
| Pseudo-second-order | $Q_{t}=\frac{Q_{e}^{2}k_{2}t}{1+Q_{e}k_{2}t}$ | Qe is the quantity of adsorbed ions after equilibration (mg/g), and K_2_ is the model rate constant (g/mg min). |
| Classic Isotherm models | | |
| Model | **Equation** | **Parameters** |
| Langmuir | $Q_{e}=\frac{Q_{max} bC_{e}}{(1+bC_{e})}$ | *C_e_* is the rest ions concentrations (mg/L), *Q_max_* is the theoritical maximum adsorption capacity (mg/g), and *b* is the Langmuir constant (L/mg) |
| Freundlich | $Q_{e}=K_{f}C_{e}^{1/n}$ | K_F_ (mg/g) is the constant of Freundlich model related to the adsorption capacity and n is the constant of Freundlich model related to the adsorption intensities |
| Dubinin–Radushkevich | $Q_{e}=Q_{m}e^{-\betaɛ^{2}}$ | β (mol^2^/KJ^2^) is the D-R constant, ɛ (KJ^2^/mol^2^) is the polanyil potential, and Q_m_ is the adsorption capacity (mg/g) |
| Advanced isotherm models | | |
| Model | **Equation** | **Parameters** |
| Monolayer model with one energy site (Model 1) | $Q=nN_{o} =\frac{nN_{M}}{1+{(\frac{C1/2}{C})}^{n}}=\frac{Q_{o}}{1+{(\frac{C1/2}{C})}^{n}}$ | Q is the adsorbed quantities in mg/g  n is the number of adsorbed ion per site  Nm is the density of the effective receptor sites (mg/g)  Q_o_ is the adsorption capacity at the saturation state in mg/g  C1/2 is the concentration of the ions at half saturation stage in mg/L  C1 and C2 are the concentrations of the ions at the half saturation stage for the first active sites and the second active sites, respectively  n1 and n2 are the adsorbed ions per site for the first active sites and the second active sites, respectively |
| Monolayer model with two energy sites (Model 2) | $Q=\frac{n_{1}N_{1M}}{1+{(\frac{C_{1}}{C})}^{n_{1}}}+\frac{n_{2}N_{2M}}{1+{(\frac{C_{2}}{C})}^{n_{2}}}$ |  |
| Double layer model with one energy site (Model 3) | $Q=Q_{o}\frac{({\frac{C}{C1/2})}^{n}+2({\frac{C}{C1/2})}^{2n}}{1+({\frac{C}{C1/2})}^{n}+({\frac{C}{C1/2})}^{2n}}$ |  |
| Double layer model with two energy sites (Model 3) | $Q=Q_{o}\frac{({\frac{C}{C1})}^{n}+2({\frac{C}{C2})}^{2n}}{1+({\frac{C}{C1})}^{n}+({\frac{C}{C2})}^{2n}}$ |  |

**Table.S2.** Comparison between the adsorption performances of the synthetic structures and other materials in literature

| Adsorbent | Q_max_ (mg/g) | References |
| --- | --- | --- |
| Sulfate ions | | |
| Microfibrillated cellulose | 7.35 | Hokkanen et al., (2017) |
| Mg–Fe-calcined LDH | 68.7 | Liu et al., (2014) |
| polypyrrole-grafted granular activated carbon | 48 | Hong et al., (2014) |
| Mg–Al LDH | 135.1 | Rahman et al., (2021) |
| Modified coconut shell fibers | 31.2 | Lima et al., (2012) |
| Cationic surfactant modified-Cu-Al LDO | 149.25 | Besharatlou et al., (2021) |
| GAC/Mg–Al LDO | 143.5 | Abushawish et al., (2023) |
| Organo-nano-clay | 38.03 | Chen et al., (2014) |
| Jordanian kaolin clay | 85.08 | Hudaib et al., (2021) |
| Polyaniline (PANI) derivative | 108.5 | Sang et al., (2013) |
| graphene oxides | 26.83 | Naghizadeh et al., (2017) |
| Carbon nanotubes | 56.94 | Alimohammadi et al., (2017) |
| Iron hydroxide | 48.03 | Gu et al., (2016) |
| Mg/GP | 234.1 | This study |
| Nitrate ions | | |
| Chitosan /ZeoliteY/ ZrO_2_ | 23.58 | Teimouri et al., (2016) |
| Al-modified biochar | 89.5 | Yin et al., (2018) |
| LTA MOFs | 50.01 | Kumar et al., (2021) |
| PEI–HCl/ cocoa shell | 86.95 | Fotsing et al., (2020) |
| MK-chitosan | 74.89 | Karthikeyan and Meenakshi., (2021) |
| Alginate@ZnFe-LDHs | 74.13 | Karthikeyan and Meenakshi., (2021) |
| HDTMA modified zeolite | 12.35 | Onyango et al., (2010) |
| Zr@CSBent composite | 23.9 | Kumar et al., (2020) |
| MXenes | 70.4 | Karthikeyan et al., (2020) |
| Biochar /Modified Zeolite | 24.45 | Wang et al., (2021) |
| Triethylamine/Giant reed | 118.9 | Ren et al., (2016) |
| Aminated alkaline lignin | 111.6 | Orlando et al., (2002) |
| Magnetic cationic hydrogel | 95.88 | Li et al., (2020) |
| Graphene | 89.9 | Ganesan et al., (2013) |
| Fe/Pd bimetal-loaded zeolite | 99.5 | He et al., (2020) |
| Amine cross-linked tea wastes | 98.72 | Qiao et al., (2019) |
| Biochar-supported polyaniline | 72 | Herath et al., (2021) |
| Mg/GP | 166.1 | This study |
